# Molecular forms of BMP15 and GDF9 in mammalian species that differ in litter size

**DOI:** 10.1038/s41598-023-49852-1

**Published:** 2023-12-16

**Authors:** Gene W. Swinerd, Abdulaziz A. Alhussini, Sarah Sczelecki, Derek Heath, Thomas D. Mueller, Kenneth P. McNatty, Janet L. Pitman

**Affiliations:** 1 School of Biological Sciences, Victoria University of Wellington, Wellington, New Zealand; 2 Department of Plant Physiology and Biophysics, Julius-Von-Sachs Institute of the University Würzburg, Würzburg, Germany

**Keywords:** Molecular biology, Proteins

## Abstract

Bone morphogenetic protein (BMP15) and growth differentiation factor (GDF9) are critical for ovarian follicular development and fertility and are associated with litter size in mammals. These proteins initially exist as pre-pro-mature proteins, that are subsequently cleaved into biologically active forms. Thus, the molecular forms of GDF9 and BMP15 may provide the key to understanding the differences in litter size determination in mammals. Herein, we compared GDF9 and BMP15 forms in mammals with high (pigs) and low to moderate (sheep) and low (red deer) ovulation-rate. In all species, oocyte lysates and secretions contained both promature and mature forms of BMP15 and GDF9. Whilst promature and mature GDF9 levels were similar between species, deer produced more BMP15 and exhibited, together with sheep, a higher promature:mature BMP15 ratio. N-linked glycosylation was prominant in proregion and mature GDF9 and in proregion BMP15 of pigs, and present in proregion GDF9 of sheep. There was no evidence of secreted native homo- or hetero-dimers although a GDF9 dimer in red deer oocyte lysate was detected. In summary, GDF9 appeared to be equally important in all species regardless of litter size, whilst BMP15 levels were highest in strict monovulatory species.

## Introduction

Litter size is dependent upon the yield of developmentally-competent oocytes ovulated at each reproductive cycle. Two members of the transforming growth factor β (TGF-β) superfamily, growth differentiation factor 9 (GDF9) and bone morphogenetic protein 15 (BMP15) have essential roles in determining ovulation quota and overall fertility in mammals^1–6^. Evidence suggests that the composition of oocyte-secreted growth factors is species-specific and leads to a set number of developmentally-competent oocytes. This includes a tightly-regulated GDF9:BM15 mRNA ratio that is unique to each species^7^, post-translational modifications that results in partially-processed forms in some species^8^ and sequence differences between species that increases receptor affinity^9^. These differences alter the potency ability of these proteins to stimulate granulosa cell proliferation in a species-specific manner^10,11^.

Members of the TGF-β superfamily are synthesised as preproproteins consisting of a pre-region, large proregion with a chaperone function, and mature domain at the carboxy-terminus^12,13^. The mature domain is preceded by a conserved RXXR motif that is cleaved by proprotein convertases such as furin^14,15^. Under the current model of protein processing, the pre-region is removed following translocation into the rough endoplasmic reticulum (RER) and the proproteins dimerise. This in turn is enzymatically-cleaved at the RXXR region leaving a mature dimer^15,16^. In many cases, even though the proregion has been enzymatically-cleaved, it may remain associated with the mature region through non-covalent interactions and continue to regulate its activity along with its cooperative interactions with other proteins^17–21^. The role of the associated proregion in regulating the activity of each protein appears to vary significantly amongst species^9,22^. Post-translational processing in the RER and Golgi apparatus, such as N- and O-glycosylation, may be essential for the bioactivity of the proteins^8,23–26^.

BMP15 and GDF9 belong to a small group within the TGF-β superfamily that lack the fourth of seven conserved cysteines within the mature peptide, and as such are unable to form a covalent bond during dimer formation^13,21^. The lack of a covalent bond within the dimer is suggested to allow for a more flexible array of possible interactions between monomer units than what may be traditionally observed with other TGF-β family members^12^. The monomers may form as non-covalent homodimers and heterodimers^18,27–29^. Numerous in vitro studies involving recombinantly produced proteins have observed homodimer, heterodimer and even multimer formations^13,18,27,28,30,31^, but not for the native forms. Moreover, the native forms of GDF9 and BMP15 are largely unknown for many species.

This study investigated the relative amounts of all molecular forms of BMP15 and GDF9 identified to be synthesised in, and secreted from, oocytes of three mammalian species that differ in litter size. The species chosen for this study were red deer, sheep and pigs with average litter sizes of 1, 1–3 and 14–22, respectively.

## Results

### Biological activity of in-house recombinant pig and red deer GDF9 and BMP15

To validate the relevance of using recombinant forms of GDF9 and BMP15 as positive controls in Western blots, their ability to bind the Type 2 receptor and furthermore, stimulate granulosa cell proliferation was assessed.

**Figure 1:**
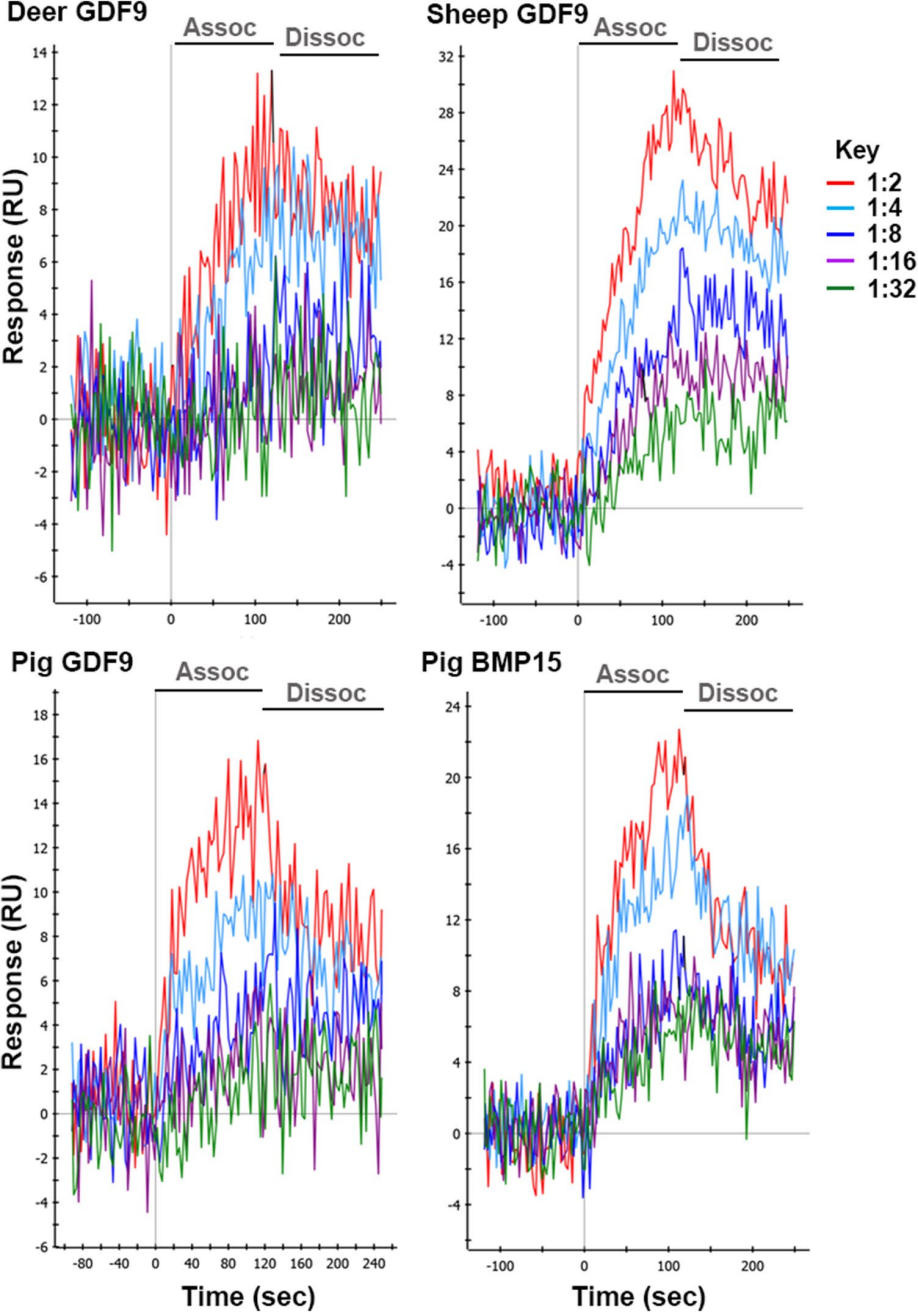
Interaction analysis of recombinant forms of cervine, ovine and porcine GDF9, and of porcine BMP15, with human (h) BMPR2 using surface plasma resonance (SPR). SPR sensograms showing the interaction of five different concentrations of recombinant red deer GDF9 (top left), sheep GDF9 (top right), pig GDF9 (bottom left) and pig BMP15 (bottom right) with hBMPR2 immobilized as ligand on the sensor surface are shown. At time point 0 (*x*-axis), the analytes were perfused over the biosensor for 120 s to monitor the association of the analytes to the hBMPR2 ectodomain protein. At time point 120 s, the sensor was perfused with running buffer to monitor the dissociation of the analytes from the Type 2 receptor. RU, resonance units.

The ability of recombinant proteins of deer BMP15 (*rec*deerBMP15), pig GDF9 (*rec*pigGDF9) and pig BMP15 (*rec*pigBMP15) to interact with a recombinant human (h)BMPR2 ectodomain is illustrated in Fig. 1. A preparation of recombinant ovine GDF9, as previously described^29^, was used as a positive control. All recombinant proteins showed a concentration-dependent association to, and dissociation from, the hBMPR2 ectodomain. However, due to the fact that the proteins were only partially purified, ligand-receptor affinities for the different GDF9 and BMP15 proteins from different species could not be calculated from the surface plasma resonance (SPR) data. However, using a sensogram of an hBMP15-hBMPR2 interaction, which employed highly purified recombinant BMP15 protein to obtain an estimate of the analyte concentration of our ovine GDF9 sample, an equilibrium binding constant K_D_ in the range of about 1 nM could be obtained.

**Figure 2:**
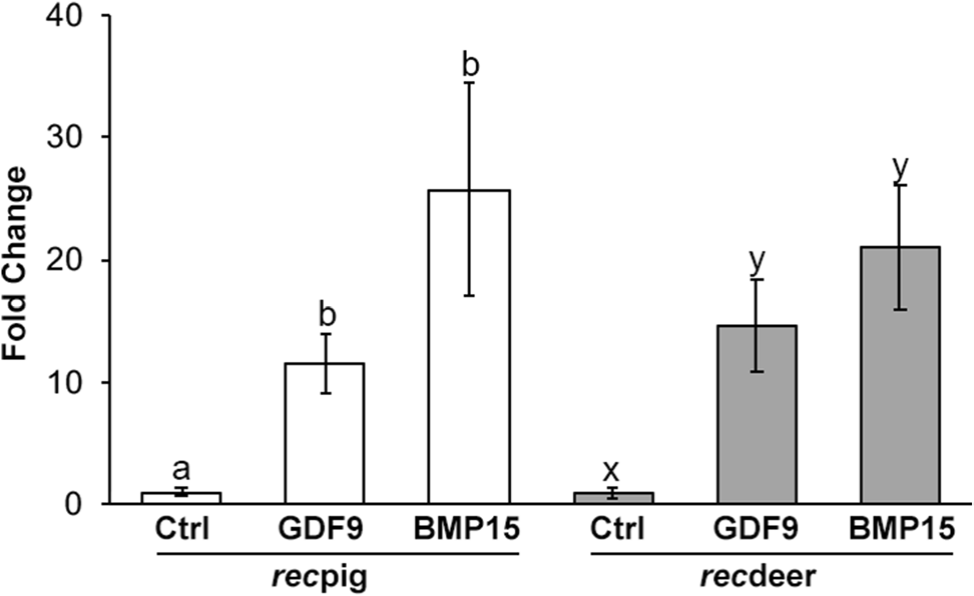
Relative proliferation rate of ovine granulosa cells following treatment with recombinant (*rec*) pig and deer GDF9 and BMP15. Mean ± SEM fold change (relative to control values) in proliferation rate was measured by a tritiated thymidine uptake assay. Different letters denote significant differences (*P* < 0.001) within each species of origin from which the recombinant proteins were produced. For each recombinant protein, different letters above the bars denote significant differences (P<0.05).

All recombinant forms were capable of stimulating ovine granulosa cells, with *rec*pigGDF9, *rec*pigBMP15, *rec*deerGDF9 and *rec*ddeerBMP15 increasing the proliferation rate at ~ 12, 26, 15 and 21-fold over that of controls (Fig. 2).

### Western blots of BMP15 produced by sheep, red deer, and pig oocytes

**Figure 3:**
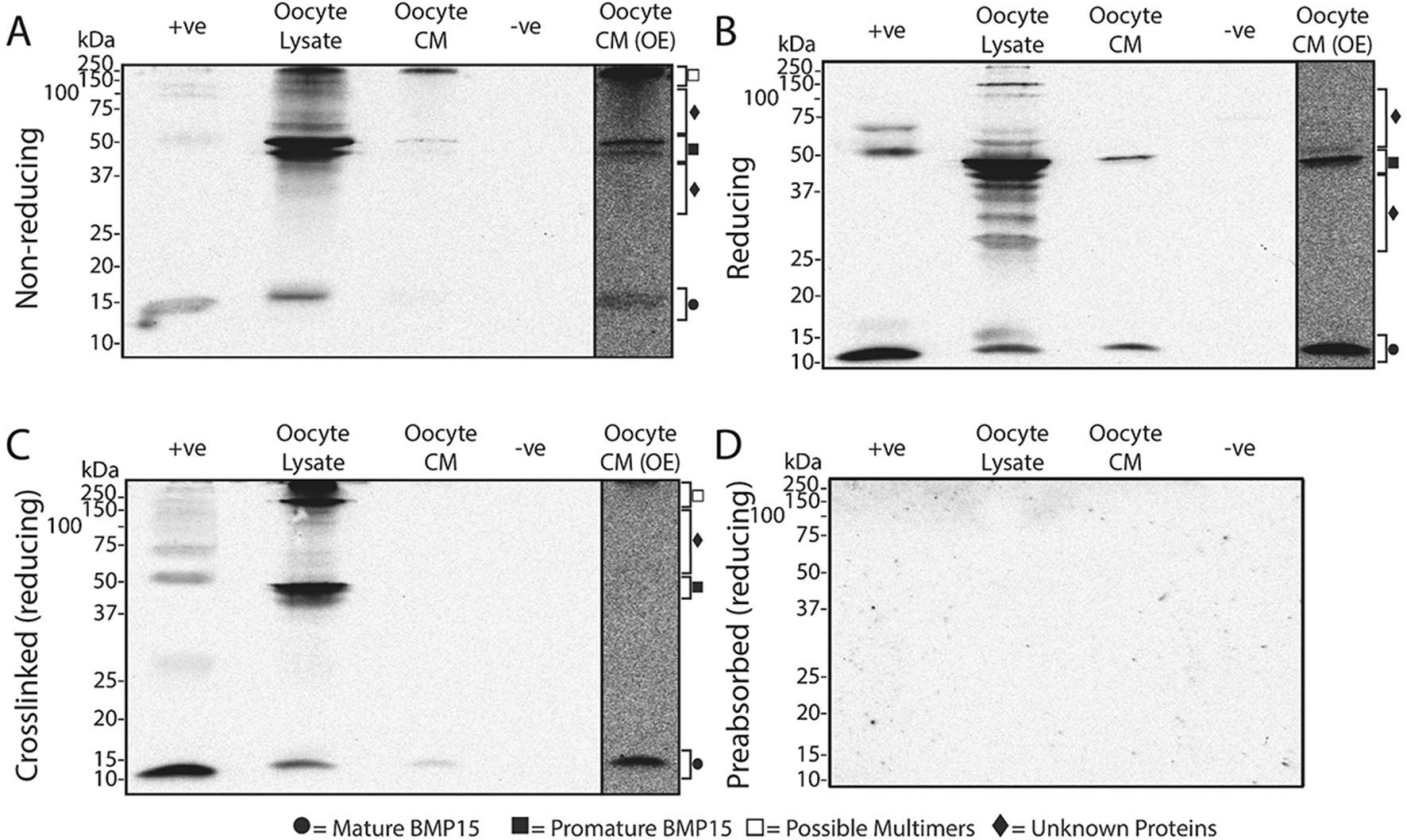
Molecular forms of BMP15 present within and secreted from sheep oocytes. Western blots were performed under non-reducing conditions (**A**), reducing conditions (**B**), and conditions where a crosslinking agent was added prior to applying reducing conditions (**C**). (**D**) is a representative blot which was stripped of all antibodies and re-probed with Mab61A which had been preabsorbed with *E.coli*-produced ovine BMP15. The lanes are as follows: + ve, recombinant HEK-293 produced pig BMP15 conditioned media (+ ve control); oocyte lysate, lysate from sheep oocytes; oocyte CM, sheep oocyte conditioned media; −ve, recombinant HEK-293 produced pig GDF9 conditioned media (−ve control); oocyte CM (OE), the oocyte CM lane (Lane 3) which has been overexposed to help clarify faint bands (*c.f.* Supplementary Fig. 1 for full overexposed blot).

**Figure 4:**
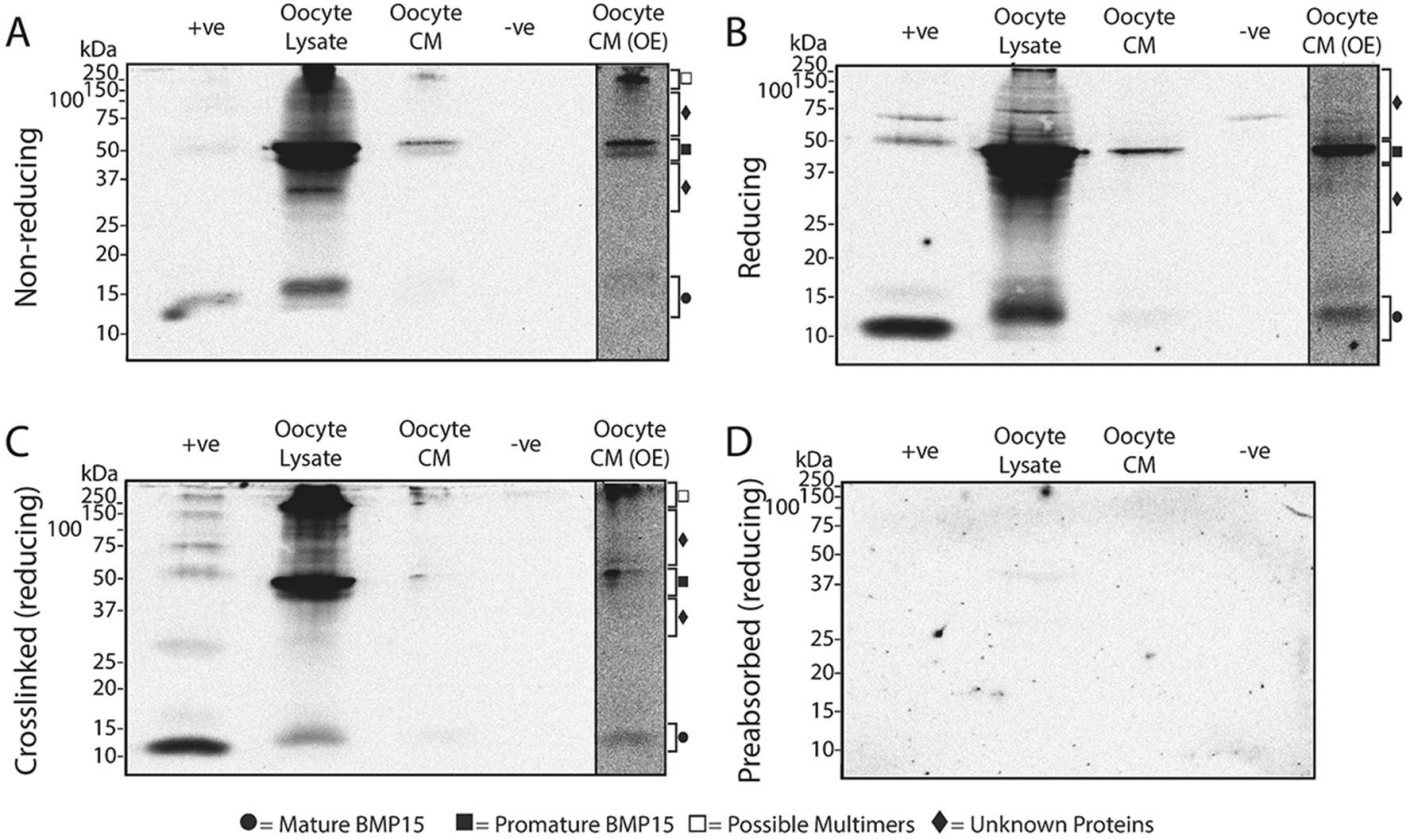
Molecular forms of BMP15 present within and secreted from red deer oocytes. Western blots were performed under non-reducing conditions (**A**), reducing conditions (**B**), and conditions where a crosslinking agent was added prior to applying reducing conditions (**C**). (**D**) is a representative blot which was stripped of all antibodies and re-probed with Mab61A which had been preabsorbed with *E.coli*-produced ovine BMP15. The lanes are as follows: + ve, recombinant HEK-293 produced pig BMP15 conditioned media (+ ve control); oocyte lysate, lysate from deer oocytes; oocyte CM, deer oocyte conditioned media, −ve, recombinant HEK-293 produced pig GDF9 conditioned media (-ve control); oocyte CM (OE), the oocyte CM lane (Lane 3) which has been overexposed to help clarify faint bands (*c.f.* Supplementary Fig. 2 for full overexposed blot).

**Figure 5:**
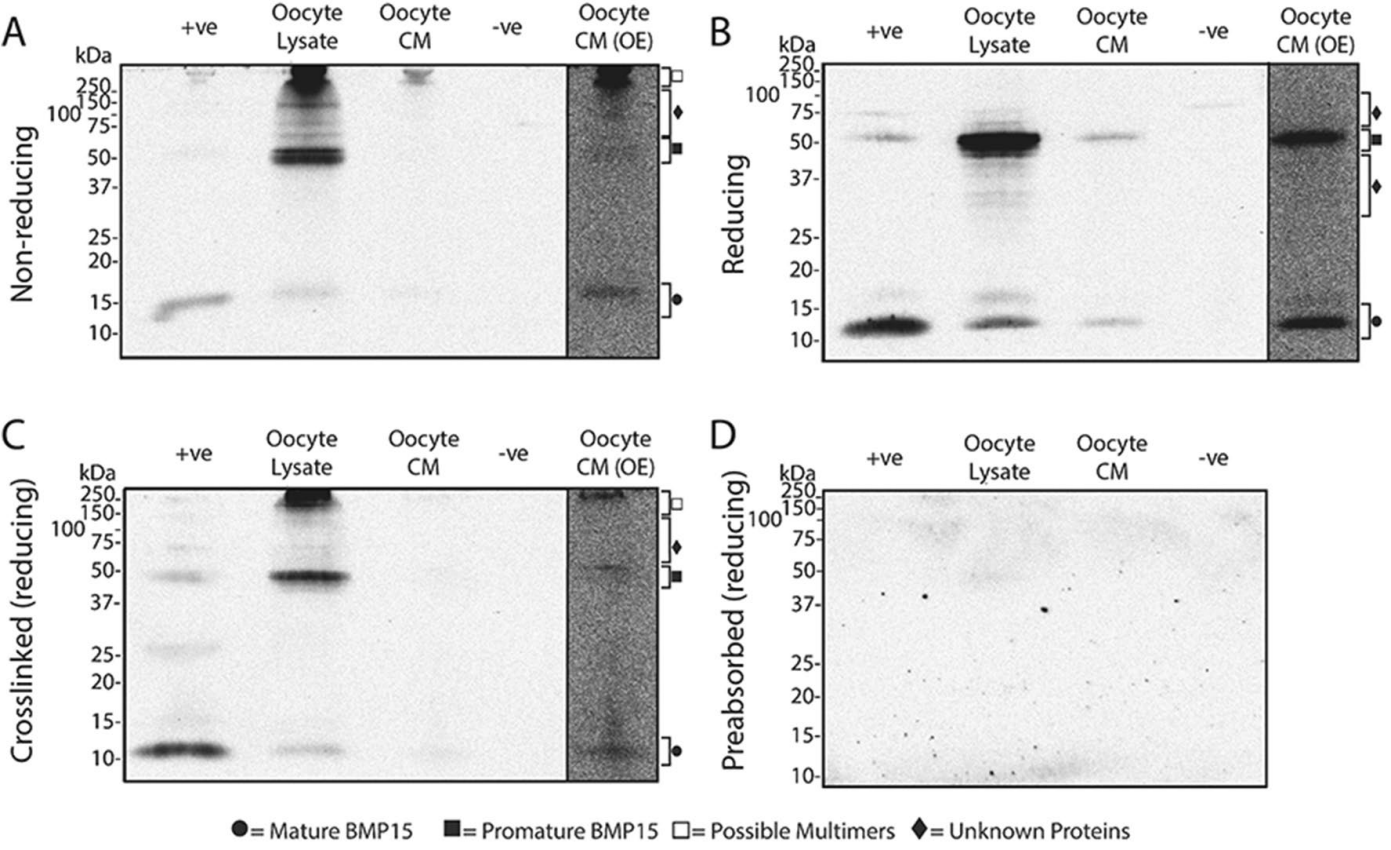
Molecular forms of BMP15 present within and secreted from pig oocytes. Western blots were performed under non-reducing conditions (**A**), reducing conditions (**B**), and conditions where a crosslinking agent was added prior to applying reducing conditions (**C**). (**D**) is a representative blot which was stripped of all antibodies and re-probed with Mab61A which had been preabsorbed with *E.coli*-produced ovine BMP15. The lanes are as follows: + ve, recombinant HEK-293 produced pig BMP15 conditioned media (+ ve control); oocyte lysate, lysate from pig oocytes; oocyte CM, pig oocyte conditioned media; −ve, recombinant HEK-293 produced pig GDF9 conditioned media (−ve control); oocyte CM (OE), the oocyte CM lane (Lane 3) which has been overexposed to help clarify faint bands (*c.f.* Supplementary Fig. 3for full overexposed blot).

The molecular forms of BMP15 present within and secreted from the oocytes of sheep, deer and pigs are depicted in Figs. 3, 4 and 5, respectively. In all figures, (A) and (B) represents samples under non-reducing and reducing conditions, respectively, (C) represents cross-linked samples under reducing conditions and (D) represents samples incubated with Mab61A antibody pre-adsorbed with *E.coli*-produced ovine BMP15.

In sheep, strong bands representing promature (~ 44–50 kDa) and mature (~ 13–15 kDa) BMP15 were detected in the oocyte lysates under all conditions tested (see Fig. 3). There was also a laddering of unknown bands below the promature band in oocyte lysates under reducing and non-reducing conditions, which disappeared following protein crosslinking. A number of unknown bands of ~ 60–150 kDa size were detected in oocyte lysates under every condition. Large-sized bands (~ 150–250 kDa) consistently appeared in oocyte lysates under non-reducing and reducing conditions when cross-linked. In the oocyte conditioned media (CM), faint bands representing promature and mature BMP15 were consistently detected under reducing and non-reducing conditions however, only bands representing mature BMP15 were detected in CM following crosslinking.

In the red deer, strong bands representing promature (~ 45–50 kDa) and faint-moderate bands representing mature (~ 13–16 kDa) BMP15 were detected in the oocyte lysate and oocyte CM under all three conditions tested (see Fig. 4). Similar to the sheep, oocyte lysates of red deer under reducing and non-reducing conditions revealed band laddering of unknown proteins smaller than promature BMP15, which reduced following protein crosslinking. There was also a smear of unknown bands between ~ 60–150 kDa in the oocyte lysate samples under each condition. Large-sized bands (~ 150–250 kDa) consistently appeared in oocyte lysates under non-reducing conditions and under reducing conditions when cross-linked, which faded under reducing conditions alone. The distinct band at ~ 30 kDa in the oocyte lysate under non-reducing conditions was not present following cross-linking and thus was not interpreted any further.

In the pig, strong bands representing promature (~ 49–52 kDa), and faint-moderate bands representing mature (~ 11–12 kDa), BMP15 were observed in oocyte lysates under all conditions (see Fig. 5). Detectable but faint bands representing promature and mature BMP15 were also observed in oocyte CM. Similar to the sheep and deer, oocyte lysates revealed band laddering of unknown proteins under the promature BMP15 band under reducing and non-reducing conditions, which was absent following protein cross-linking. A number of undefined bands appeared between ~ 60-150 kDa in the oocyte lysate samples under each condition. There were bands between ~ 150–250 kDa in the oocyte lysate and oocyte CM samples under non-reducing conditions and following crosslinking that were significantly reduced or absent under reducing conditions alone.

Crosslinking of *rec*pigBMP15 (positive control) resulted in the appearance of a ~ 28–30 kDa band under reducing conditions in all replicate blots performed for all four species (Figs. 3C, 4C and 5C). This band size is consistent with a mature BMP dimer.

Preabsorption of Mab61A with *E.coli*-produced ovine BMP15 resulted in no visible bands under reducing conditions (Figs. 3D, 4D and 5D), supporting the assumption that the bands observed are due to the specific binding of the antibody to BMP15. A similar lack of bands was observed in samples under non-reducing conditions and in cross-linked samples under reducing conditions when incubated with pre-adsorbed antibody (data not shown).

### Comparisons of molecular forms of BMP15 in sheep, red deer, and pigs

**Figure 6:**
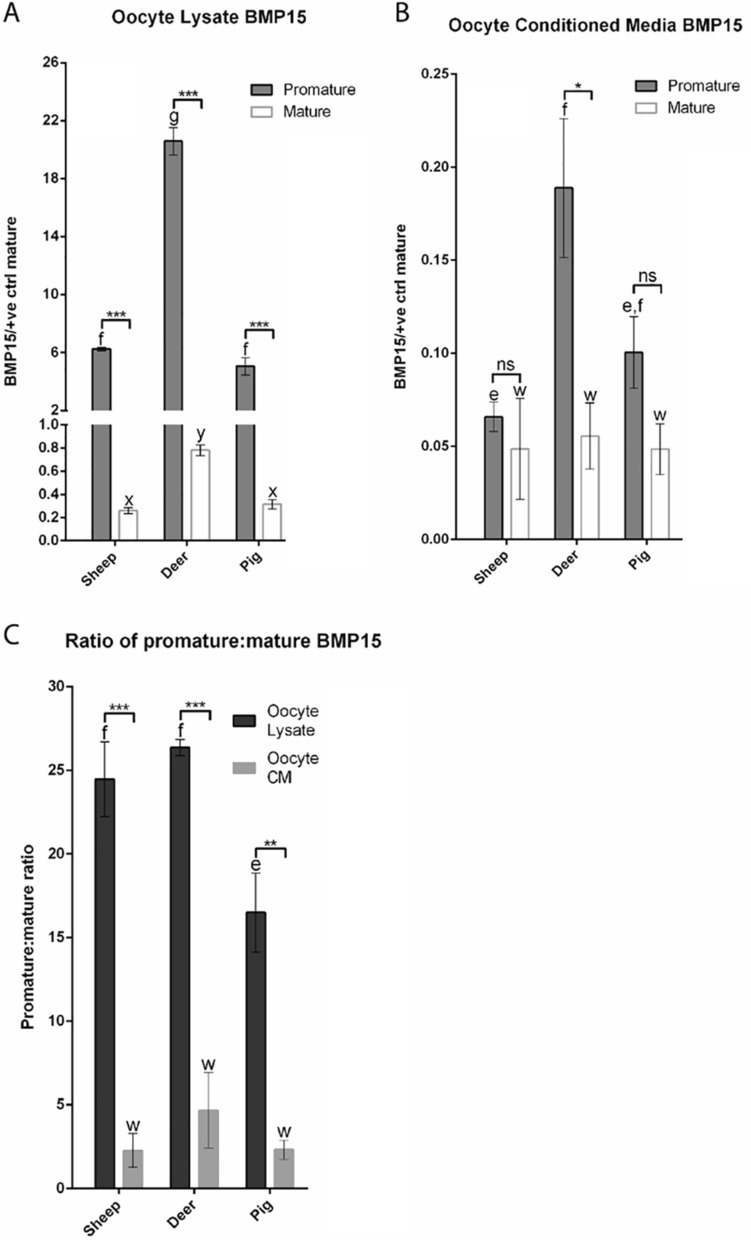
Densitometry analyses of BMP15 bands under reducing conditions. (**A**) and (**B**) represent the mean normalised density values of the promature and mature *BMP15* bands detected in the oocyte lysates (**A**) and oocyte conditioned media (CM) (**B**) of each species. (**C**) represents the mean promature:mature ratios detected in the oocyte lysates and oocyte CM of each species. In each graph, different letters above columns of the same shade indicates a significant difference between the species (*P* ≤ 0.05), while identical letters indicated no significant difference (*P* > 0.05). Brackets are used to indicate differences between the two different shaded columns within each species (* = *P* ≤ 0.05, *** = *P* ≤ 0.001, ns = not significant). The vertical error bars represent ± SEM.

Relative amounts of promature and mature BMP15 within each species were calculated by densitometry and normalised against oocyte number and the mature band of *rec*pigBMP15 in each blot. The relative amounts of promature and mature BMP15 in oocyte lysate and CM in each species are depicted in Figs. 6A,B, respectively. The ratios of promature:mature BMP15 in oocyte lysate and oocyte CM in each species are depicted in Fig. 6C.

The oocyte lysates of all species contained more (*p* < 0.001) promature BMP15 than mature BMP15 (Fig. 6A). When comparing the relative amounts between species, the oocyte lysate of red deer contained more promature (*p* < 0.05) and mature (*p* < 0.05) BMP15, compared to the other species. Thus, the relative amounts of promature BMP15 in the oocyte lysates of red deer, sheep and pig were 20.5, 6.2 and 5.2, respectively. The relative amounts of mature BMP15 in the oocyte lysates of red deer, sheep and pig were 0.78, 0.26 and 0.31, respectively.

The deer was the only species in which oocyte CM contained more (*p* < 0.05) promature than mature BMP15 (Fig. 6B). Moreover, the relative amount of promature BMP15 in oocyte CM of deer was more (*p* < 0.05) than that in any other species. This was followed by the sheep and pig, in which oocyte CM that contained similar amounts of promature BMP15, and the relative amounts of mature BMP15 were similar between species. Thus, the relative amounts of promature BMP15 in the oocyte CM of red deer, sheep and pig were 0.19, 0.07 and 0.1, respectively. The relative amounts of mature BMP15 in the oocyte CM of red deer, sheep and pigs were 0.06, 0.05 and 0.05, respectively.

When the ratios of promature:mature BMP15 were compared as depicted in Fig. 6C, the oocyte lysate contained markedly higher (*p* < 0.001) ratios of promature:mature BMP15, compared to oocyte CM, in all species. In the oocyte lysate, the promature:mature BMP15 ratio was higher (P < 0.05) in both the red deer (26.4) and sheep (24.5) than the pig (16.5). In contrast, the oocyte CM contained similar ratios of promature:mature BMP15 in the deer (4.7), sheep (2.3) and pig (2.3).

### Western blots of GDF9 produced by sheep, red deer, and pig oocytes

**Figure 7:**
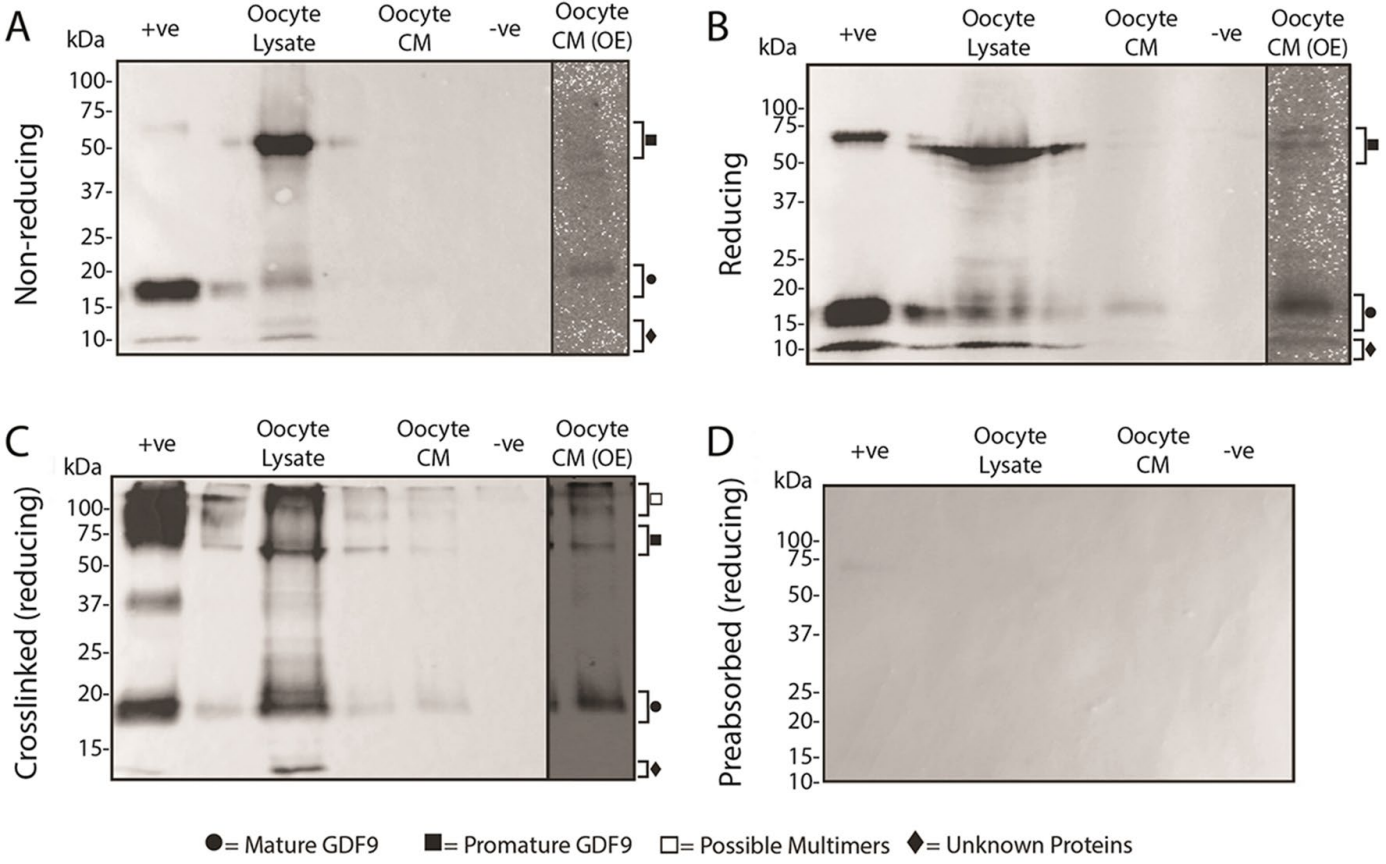
Molecular forms of GDF9 present within and secreted from sheep oocytes. Western blots were performed under non-reducing conditions (**A**), reducing conditions (**B**), and conditions where a crosslinking agent was added prior to applying reducing conditions (**C**). (**D**) is a representative blot which was stripped of all antibodies and re-probed with Mab37A which had been preabsorbed with *E.coli*-produced ovine GDF9. The lanes are as follows: + ve, recombinant HEK-293 produced pig GDF9 conditioned media (+ ve control); oocyte lysate, lysate from sheep oocytes; oocyte CM, sheep oocyte conditioned media; −ve, recombinant HEK-293 produced pig BMP15 conditioned media (−ve control); oocyte CM (OE), the oocyte CM lane (Lane 3) which has been overexposed to help clarify faint bands (*c.f.* Supplementary Fig. 4for full overexposed blot).

**Figure 8:**
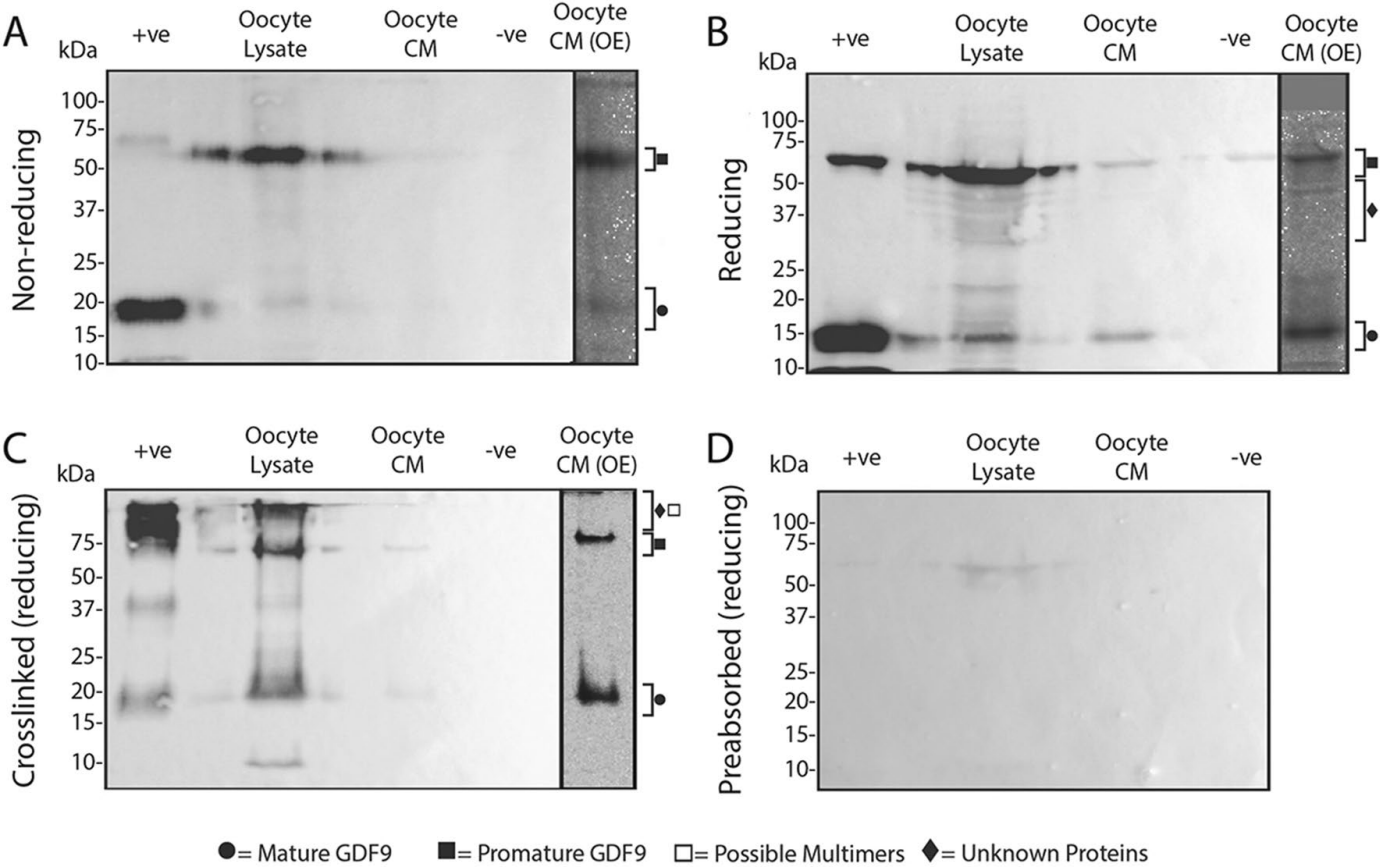
Molecular forms of GDF9 present within and secreted from red deer oocytes. Western blots were performed under non-reducing conditions (**A**), reducing conditions (**B**), and conditions where a crosslinking agent was added prior to applying reducing conditions (**C**). (**D**) is a representative blot which was stripped of all antibodies and re-probed with Mab37A which had been preabsorbed with *E.coli*-produced ovine GDF9. The lanes are as follows: + ve, recombinant HEK-293 produced pig GDF9 conditioned media (+ ve control); oocyte lysate, lysate from deer oocytes; oocyte CM, deer oocyte conditioned media; −ve, recombinant HEK-293 produced pig BMP15 conditioned media (−ve control); oocyte CM (OE), the oocyte CM lane (Lane 3) which has been overexposed to help clarify faint bands (*c.f.* Supplementary Fig. 5for full overexposed blot).

**Figure 9:**
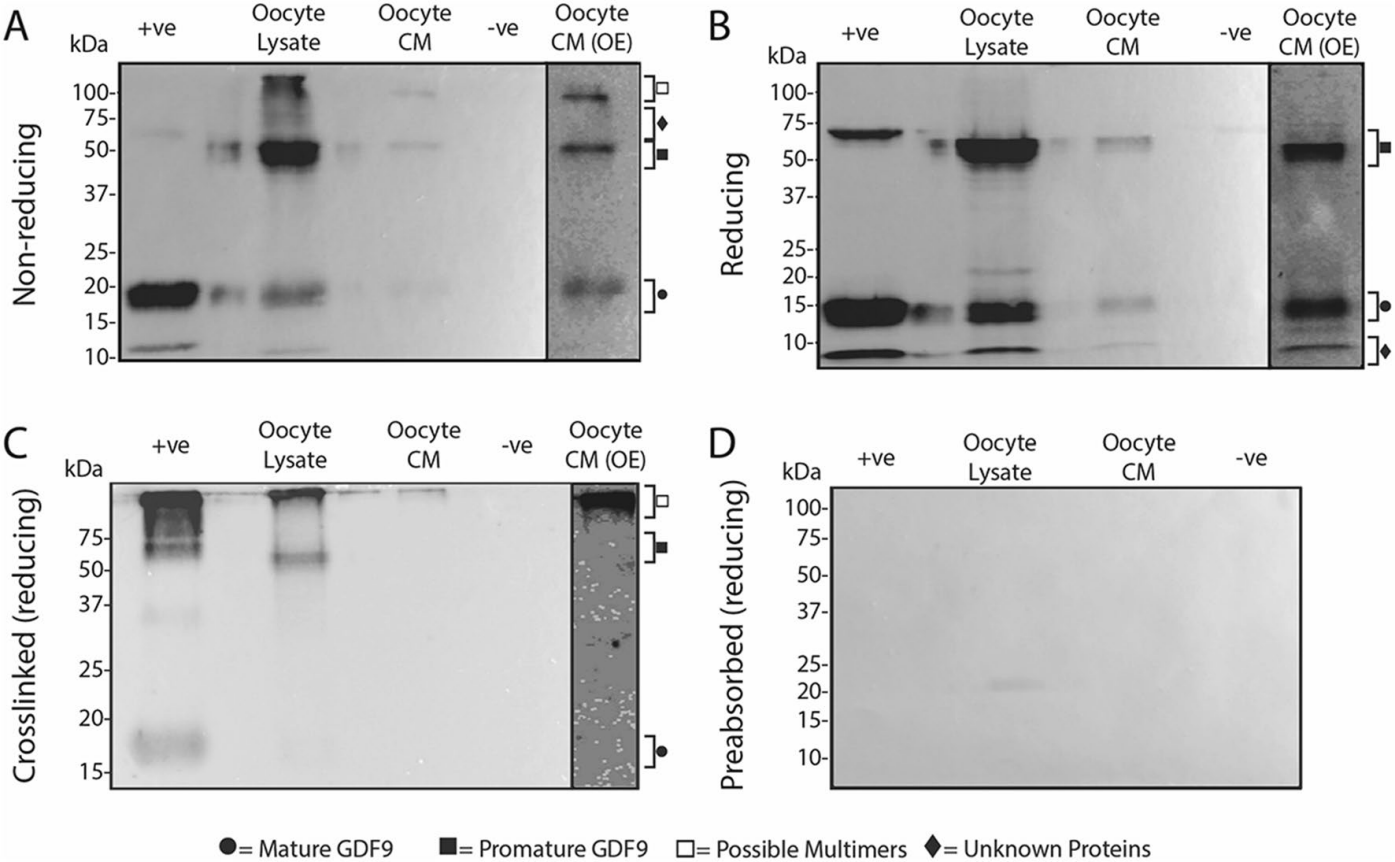
Molecular forms of GDF9 present within and secreted from pig oocytes. Western blots were performed under non-reducing conditions (**A**), reducing conditions (**B**), and conditions where a crosslinking agent was added prior to applying reducing conditions (**C**). (**D**) is a representative blot which was stripped of all antibodies and re-probed with Mab37A which had been preabsorbed with *E.coli*-produced ovine GDF9. The lanes are as follows: + ve, recombinant HEK-293 produced pig GDF9 conditioned media (+ ve control); oocyte lysate, lysate from pig oocytes; oocyte CM, pig oocyte conditioned media; −ve, recombinant HEK-293 produced pig BMP15 conditioned media (−ve control); oocyte CM (OE), the oocyte CM lane (Lane 3) which has been overexposed to help clarify faint bands (*c.f.* Supplementary Fig. 6 for full overexposed blot).

The molecular forms of GDF9 present within and secreted from the oocytes of sheep, red deer and pigs are depicted in Figs. 7, 8 and 9, respectively. In all figures, (A) and (B) represents samples under non-reducing and reducing conditions, respectively, (C) represents cross-linked samples under reducing conditions and (D) represents samples incubated with Mab61A antibody pre-adsorbed with *E.coli*-produced ovine GDF9.

In sheep, bands representing promature (~ 55–60 kDa) and mature (~ 16–18 kDa) GDF9 were detected in oocyte lysates under all three conditions tested (see Fig. 7). In oocyte CM, faint promature and mature GDF9 bands were detected with long exposure times under reducing conditions regardless of crosslinking, however only mature GDF9 was detected with long exposure times under non-reducing conditions. Crosslinking of the oocyte lysate and oocyte CM samples resulted in the detection of both promature and mature GDF9 as well as a number of high molecular weight bands (> 100 kDa).

In red deer, bands representing promature (~ 63–67 kDa) and mature (~ 14–19 kDa) GDF9 were detected in the oocyte lysate and oocyte CM under all three conditions tested (see Fig. 8). Similar to the sheep samples, oocyte lysates of the deer exhibited high molecular weight bands (> 100 kDa) following crosslinking. The faint band at ~ 37 kDa (similar size to a mature GDF9 dimer) in the oocyte lysate sample under non-reducing conditions was absent in the other replicate blots. In the oocyte lysate sample under reducing condition, there was a laddering of unknown bands below the promature band.

In the pig, bands representing promature (~ 55–62 kDa) and mature (~ 13–16 kDa) GDF9 were detected in the oocyte lysate and oocyte CM under non-reducing and reducing conditions (see Fig. 9). Following crosslinking, only the promature band in the oocyte lysate was detected. Under non-reducing conditions and after protein crosslinking, a high molecular weight (> 100 kDa) band was present in both the oocyte lysate and oocyte CM samples.

Crosslinking of *rec*pigGDF9 (positive control) resulted in the appearance of a ~ 37 kDa band under reducing conditions in all replicate blots performed for all four species (Figs. 7C, 8C and 9C). This band size is consistent with a mature GDF9 dimer.

Preabsorption of Mab37A with *E.coli*-produced ovine GDF9 resulted in no visible bands under reducing conditions (Figs. 7D, 8D and 9D), supporting the assumption that the bands observed were due to the specific binding of the antibody to GDF9. There was a similar absence of bands in samples under non-reducing conditions and in cross-linked samples under reducing conditions when incubated with pre-adsorbed antibody (data not shown).

### Comparisons of molecular forms of GDF9 in sheep, red deer, and pigs

**Figure 10:**
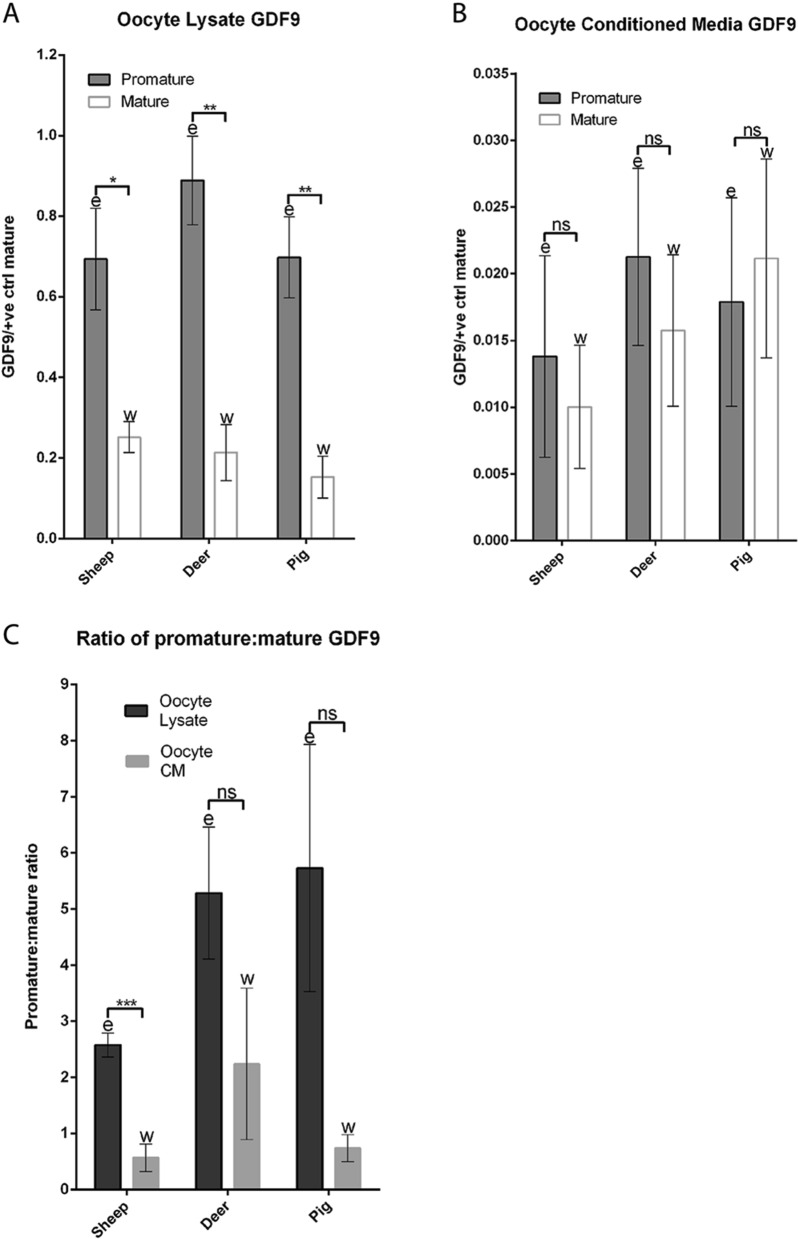
Densitometry analyses of GDF9 bands under reducing conditions. (**A**) and (**B**) represent the mean normalised density values of the promature and mature GDF9 bands detected in the oocyte lysates (**A**) and oocyte conditioned media (CM) (**B**) of each species. (**C**) represents the mean promature:mature ratios detected in the oocyte lysates and oocyte conditioned media of each species. In each graph, different letters above columns of the same shade indicates a significant difference between the species (*P* ≤ 0.05), while identical letters indicate no significant difference (*P* > 0.05). Brackets are used to indicate differences between the two different shaded columns within each species (* = *P* ≤ 0.05, *** = *P* ≤ 0.001, ns = not significant). The vertical error bars represent ± SEM.

Relative amounts of promature and mature GDF9 within each species were calculated by densitometry and normalised against oocyte number and the mature band of *rec*pigGDF9 in each blot. The relative amounts of promature and mature GDF9 in oocyte lysate and oocyte CM in each species are depicted in Figs. 10A,B. The ratios of promature:mature GDF9 in oocyte lysate and oocyte CM in each species are depicted in Fig. 10C.

The oocyte lysates of the sheep, red deer and pig contained more (P < 0.001) promature GDF9 than mature GDF9 (Fig. 10A). When comparing the relative amounts between species, the oocyte lysates contained similar relative amounts of promature and mature GDF9 in the sheep, deer and pig. Thus, the relative amounts of promature GDF9 in the oocyte lysates of the sheep, deer and pig were 0.69, 0.89 and 0.70, respectively. The relative amounts of mature GDF9 in the oocyte lysates of the sheep, deer and pig were 0.25, 0.21 and 0.15, respectively.

The oocyte CM of the sheep, deer and pig contained similar amounts of promature (0.014, 0.021 and 0.018, respectively) and mature (0.010, 0.015 and 0.021, respectively) GDF9 (Fig. 10B).

When the ratios of promature:mature GDF9 were compared as depicted in Fig. 10C, the sheep was the only species in which the promature:mature GDF9 ratio was higher (*p* < 0.001) in oocyte lysate compared to oocyte CM. It should be noted that the trend of higher promature:mature ratios in the red deer and pig was not significant due to large variation between samples and low replicate numbers (n = 3). When the promature:mature GDF9 ratios were compared between sheep, red deer, and pigs, there were no differences in oocyte lysates (2.58, 5.28 and 5.73 respectively), or the oocyte CM (0.57, 2.24 and 0.74 respectively).

### Glycosylation of BMP15 and GDF9 in sheep, deer, and pigs

**Figure 11:**
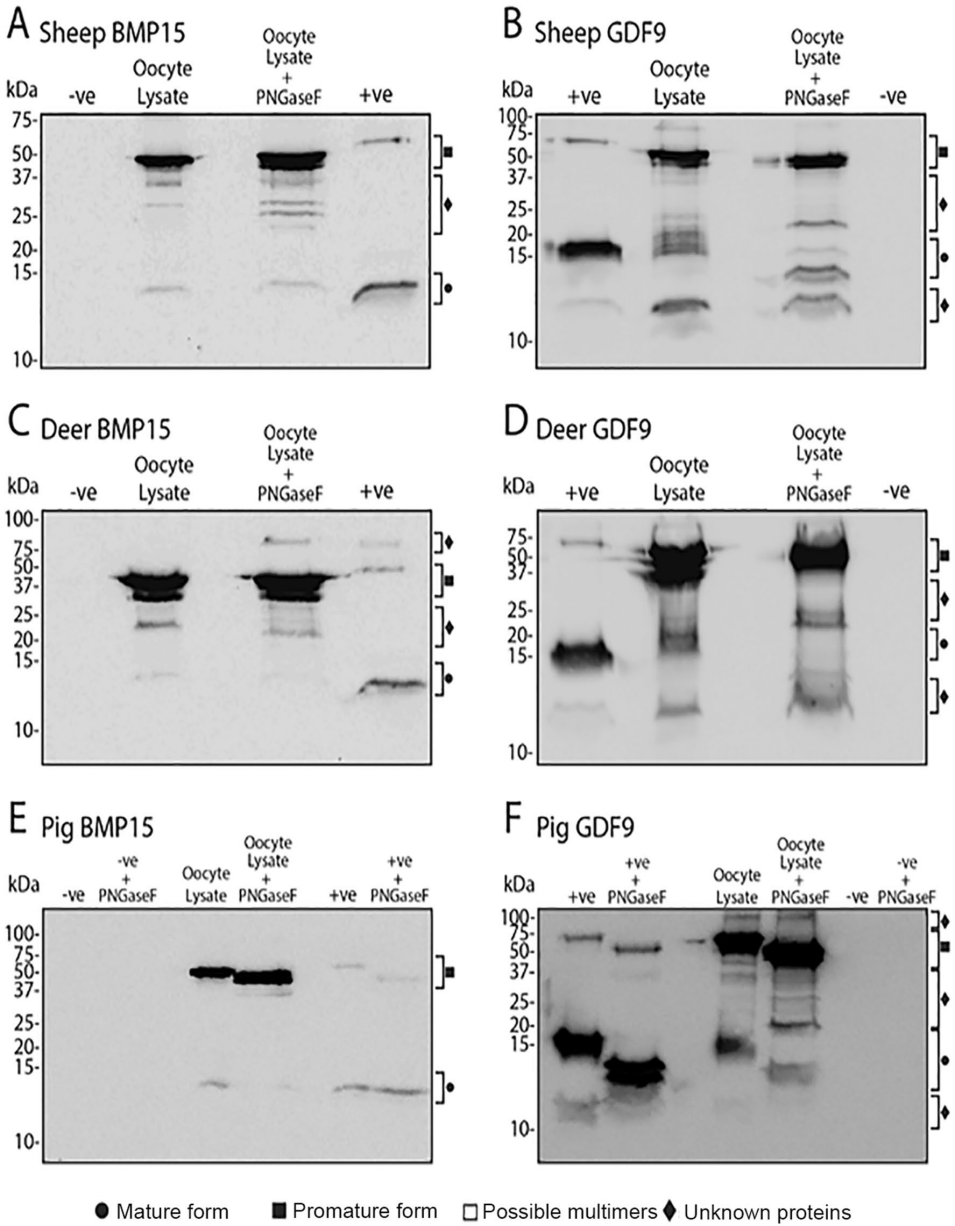
Molecular forms of BMP15 and GDF9 of sheep, red deer and pigs in the absence or presence of an N-linked deglycosylation agent. Western blots were performed under reducing conditions using the oocyte lysates of sheep (**A** and **B**), red deer (**C** and **D**), and pig (**E** and **F**). Blots (**A**, **C** and **E**) were visualised for BMP15 using Mab61A, while blots (**B**, **D** and **F**) were visualised for GDF9 using Mab37A. The lanes are as follows: + ve, recombinant HEK293-produced pig BMP15 (blots A, C and E) or GDF9 (blots **B**, **D**, and **F**) conditioned media (+ ve control); oocyte lysate, lysate from oocytes; + PNGaseF, indicates that the sample was treated with the endoglycosidase PNGase F; −ve, recombinant HEK293-produced pig GDF9 (blots **A**, **C** and **E**) or BMP15 (blots **B**, **D**, and **F**) conditioned media (−ve control).

The degree of glycosylation of promature and mature forms of BMP15 and GDF9 in the oocyte lysates of sheep, deer, and pigs both are depicted in Fig. 11. The approximate shift in the molecular weights of promature and mature protein bands in sheep, deer and pigs following treatment with PNGase F are summarised in Table 1. Treatment with PNGase F caused no change in band size for any molecular forms of BMP15 in sheep and red deer (Fig. 11A,C). In contrast, following PNGase F treatment, the band representing pig promature BMP15 was reduced by ~ 7 kDa, while mature pig BMP15 remained unchanged (Fig. 11E). This observation was supported by results in PNGase F-treated *rec*pigBMP15, whereby a similar size reduction was observed in the promature protein (Fig. 11E).

**Table 1:** Changes in protein mass (kDa) of sheep, red deer and pig BMP15 and GDF9 following deglycosylation treatment. Changes in mass (kDa) of native forms of sheep, red deer and pig BMP15 and GDF9, and recombinant forms of red deer and pig BMP15 and GDF9 following treatment with PNGase F to remove any N-linked glycans were estimated by Western blotting (*c.f.* Fig.11). The values entered represent the estimated mass of de-glycosylated minus glycosylated protein.

	Oocyte lysate			Recombinant protein	
	Sheep	Deer	Pig	Deer	Pig
Promature BMP15	0	0	− 7	0	− 7
Mature BMP15	0	0	0	0	0
Promature GDF9	− 3	0	− 10	0	− 10
Mature GDF9	− 3–5	0	− 2	0	− 2

Treatment with PNGase F resulted in a ~ 3 kDa reduction in the band representing mature ovine GDF9, and it was likely that the reduction in band size observed for promature ovine GDF9 was due solely to the differences in the mature form (Fig. 11B). A new unknown band appeared at ~ 22 kDa and laddering near the promature and mature bands in the glycosylated samples appeared to fade following treatment with PNGase F (Fig. 11B). In deer, treatment with PNGase F caused no change in the mass of promature or mature GDF9 (Fig. 11D). Band laddering directly under the promature band in the glycosylated samples disappeared after treatment with PNGase F (Fig. 11D). In pig, a marked decrease in band sizes was observed in promature GDF9 (10 kDa), of which only approximately 2 kDa could be attributed to mass loss in the mature protein (Fig. 11F). This was supported by similar results in the promature and mature forms of *rec*pigGDF9 (Fig. 11F). A new unknown band appeared at ~ 20 kDa, and unlike the sheep and red deer samples, there was no change in the laddering of the bands following treatment with PNGase F (Fig. 11F).

## Discussion

This study compared the native oocyte-produced molecular forms of GDF9 and BMP15 in sheep, red deer and pigs. While the intra-oocyte and oocyte-secreted forms of BMP15 and GDF9 in sheep have been reported previously^10,29^, the present study provided novel data on two new species that have divergent litter sizes (i.e. pigs and red deer). Moreover, cross-linking and de-glycosylation experiments reported herein provide interesting new insights into differences in the molecular forms between all three species tested. Both promature and mature forms of BMP15 and GDF9 were successfully detected within the oocyte lysates and CM of sheep, red deer and pigs. It should be noted that there are limitations to this study which include the semi-quantitative (not fully quantitative) nature of Western blotting. The assumption must also be made that the antibodies utilised in this study bound to their antigens in an equal manner, regardless of species differences. Whilst the capabilities of both antibodies to detect differences in amounts of GDF9 and BMP15 was successfully established using serial dilutions of recombinant proteins and/or oocytes (Supplemental Figs. 1 and 2)), caution should be taken for interpreting subtle between-species differences.

Initially, evidence suggested that BMP15 had a lower importance than GDF9 on follicular development in mammalian species with high litter sizes^32^. However, pig oocytes have been reported to express appreciable levels of *BMP15* mRNA and BMP15 protein^7,33,34^, therefore this may only be true for rodent species. This is supported by results from this study whereby unlike rats, promature and mature BMP15 were detected alongside GDF9 in oocyte lysates and CM of pigs. In fact, BMP15 has been reported to improve porcine oocyte maturation in vitro, with roles including the prevention of cumulus cell apoptosis^33,35^. Interestingly, we reported that pig granulosa cells also express *GDF9* mRNA to a level within an entire follicle that is relative to half that expressed by the oocyte^11^. This corrects the low intra-oocyte *GDF9:BMP15* ratio initially reported for the pig^7^ and supported our hypothesis that the intra-follicular ratio of GDF9:BMP15 is directly correlated to litter size in mammals.

The promature forms of BMP15 and GDF9 that were present in oocyte lysates and CM appeared to be primarily in an uncleaved form as evidenced by a promature band under reducing conditions. Uncleaved promature forms were expected within the oocyte of all species as current evidence suggests both proteins are produced in a similar manner to that of all TGF-β proteins. That is, they enter the RER as uncleaved promature proteins^12,36^. However, the observation of promature BMP15 and GDF9 with uncleaved pro-regions in oocyte CM of sheep, deer and pigs was more noteworthy. This is because the mature region of the TGFβ superfamily members is considered biologically active, and removal of the proregion from the mature peptide usually occurs intracellularly before secretion from the oocyte^13,37^. However, it is not uncommon for a cleaved proregion of TGFβ superfamily proteins to remain non-covalently associated with their corresponding mature peptides in a latent complex^37^. While the results from this current study do not rule out the possibility that some promature BMP15 and GDF9 is secreted in a semi-processed non-covalently associated state, there is strong evidence that a significant portion is in an unprocessed form due to the presence of bands under reducing conditions. While secreted uncleaved promature forms are not common amongst the other TGFβ superfamily members, a number of studies using cell lines producing recombinant human, sheep or mouse BMP15 have observed uncleaved promature forms in the CM^8,18,38–41^. The presence of unprocessed promature BMP15 in the oocyte CM of sheep, deer and pigs suggests that it may have a biological function. Extracellularly, the proregions of TGF-β superfamily proteins are reported to act as chaperones for aiding and regulating the delivery of the mature ligand to their target cells and may regulate dimer and receptor interactions^18,28,29,42,43^. Therefore, the presence of uncleaved promature BMP15 and GDF9 may allow each species to have additional species-specific extracellular mechanisms for regulation of BMP15 signalling.

In the three species tested in this study, there was no evidence that secreted forms of mature or promature homo- (GDF9/GDF9 or BMP15/BMP15) or hetero- (BMP15/GDF9) dimers exist. Indeed, a band representing a dimer size (~ 37KDa) was observed after crosslinking in lysed oocytes of red deer but not in secreted media. As mentioned earlier, BMP15 and GDF9 lack the seventh cysteine residue that forms the disulphide bridge between two monomer subunits, presumably allowing for more flexible interactions between the monomer units^12,13^. While numerous studies have identified homodimers and heterodimers in recombinantly-produced forms of BMP15 and GDF9 following chemical crosslinking^8,13,18,28,30,31,44^ and indeed also in this study, there is currently no evidence to suggest these forms are present in follicular fluid in vivo. The lack of dimers observed in native secreted forms may be due to their presence being below the detectable threshold. To mitigate this, we lengthened exposure times to encourage the detection of faint bands. Alternatively, it is possible that the antibodies used could not detect the native dimers, however this is unlikely as mature BMP15 and GDF9 homodimers were detected in respective positive controls of their recombinant forms after crosslinking. Finally, it is possible that native dimers are not stable under the collection conditions used and separated prior to crosslinking. Despite all these limitations, the absence of any dimers under the conditions used in this study, together with the absence of any other evidence of native dimer formation in the literature poses an interesting question about the differences between recombinant and native forms.

Covalent, and possibly non-covalent, multimers containing BMP15 were observed in oocyte lysates and CM of all three species. These were evident through the presence of prominent 150–250 kDa bands under crosslinking and/or non-reducing conditions. Furthermore, based on alignment of bands, multimers of similar molecular weights were detected in oocyte lysates and CM of all species in both BMP15 and GDF9-labelled blots. There was insufficient evidence to determine whether these multimers contained more than one BMP15 and GDF9 monomer each; it is possible that they contained multiple BMP15 and/or GDF9 molecules along with other unknown proteins. The broadness of the multimer band suggests there may be a range of different multimeric compounds present. Similar bands have been observed in previous studies using recombinant proteins^18,40,41,45^.

Both BMP15 and GDF9 have been reported to exhibit glycosylation sites in both their promature and mature regions^36,46^. N-linked glycosylation sites on the proregion of TGFβ1 are essential for both secretion and bioactivity of the protein^24^, however, their importance in BMP15 or GDF9 is unknown. De-glycosylation of lysates of sheep, deer, and pig oocytes revealed some interesting species differences in the degree of N-glycosylation of each protein. The only species in which N-linked glycosylation was observed in the proregion of BMP15 was the pig, and there was no N-linked glycosylation within the mature BMP15 protein in any species tested. The de-glycosylation of recombinant pig promature and mature BMP15 protein supported these results. N-linked glycosylation appeared to be more common in GDF9 as both sheep and pig mature GDF9 and pig promature GDF9 exhibited N-glycosylation sites. These results were supported by that observed using recombinant pig GDF9 protein. It is likely that the change in mass of sheep promature GDF9 following PNGase F was due to the removal of the N-glycosylation sites on the mature region. No molecular forms of red deer GDF9 or BMP15 were N-glycosylated. It has been previously suggested that the level of N-linked glycosylation in BMP15 and GDF9 may have an important role in determining the bioactivity of the proteins^8,26,38^. Thus, it is interesting that the polyovulatory (i.e. pig) and strict monoovulatory (i.e. red deer) species differed in the degree of N-linked glycosylation in the proregions of GDF9 and BMP15. The polyovulatory species exhibited a high degree of N-linked glycosylation, particularly in the proregions of both BMP15 and GDF9.

The relative amounts of promature and mature BMP15 and GDF9 detected under reducing conditions were calculated by generating normalised densitometry values for each band. While the method used may allow within reason, comparisons to be made between species for either BMP15 or GDF9, the density values of BMP15 cannot be directly compared with the density values of GDF9. The finding that lysates of deer oocytes had relatively high levels of promature and mature BMP15 supports previously reported gene expression data in these species^7^ and the hypothesis that BMP15 is essential for low ovulation rate species^32,47,48^. However, similar to gene expression findings in this species^7^, moderate BMP15 levels were detected also in pig oocytes and in media in which pig oocytes were incubated. Therefore, despite both the oocyte (this study) and granulosa cells^11^ contributing to the intrafollicular GDF9 concentrations, this study demonstrates that unlike in rodents^8,10^, considerable amounts of BMP15 are also present within the pig follicle.

As expected, the promature:mature ratio of GDF9 and BMP15 was lower in CM supporting previous suggestions that the promature form may be less readily secreted than the processed mature form^13^ or that processing of the promature protein occurs more rapidly outside of the cell. Another possibility is that extracellular protease activity under in vitro conditions led to increased promature processing. While protease inhibitors were added after the incubation of the oocytes, protease activity in the CM may still have occurred during the incubation period. This possibility is supported by studies that revealed that only the unprocessed promature form was present in follicular fluid of sheep^39^ in comparison to the processed and unprocessed forms detected in media from in-vitro culturing of sheep oocytes^10^. Herein, the promature:mature GDF9 ratios in the oocyte lysates did not differ between species. This may be partially explained by the fact each species has the same RHRR sequence in the furin-like protease cleavage region of the GDF9 molecule. In contrast, the promature:mature BMP15 ratio in oocyte lysates differed between the low and high ovulation rate species with the ratio being markedly higher in the low ovulation rate species. The lower ratio in the species with large litter sizes does not conform well with a number of studies whereby mutations that lowered promature BMP15 processing (i.e. increased the promature:mature ratio) led to higher fertility^8,13,48^. The promature:mature BMP15 ratios observed may be partially explained by the fact that both pigs and rats have unique residues in the XX region of the RXXR furin-like protease cleavage region while sheep and deer both share the RRAR cleavage sequence, resulting in different cleavage rates.

In summary, this study suggests that the amount of GDF9 produced within and secreted from oocytes does not differ between species. This supports previous evidence suggesting that GDF9 is equally important across all species, regardless of litter size. Nonetheless, inter-species differences exist in the degree of N-linked glycosylation of GDF9 perhaps implying that pig, and to a lesser extent sheep, GDF9 has an increased bioactivity. Species differences are most evident when comparing BMP15 in regards to production, secretion and degree of N-linked glycosylation. In general, this study together with recent results of other studies strengthens the hypothesis that lower BMP15 levels, either alone (rodents)^8,10,11^ or as a ratio against GDF9 (pigs; this study)^11^, are associated with a higher litter size. The lower promature:mature ratio of BMP15 and the presence of N-linked glycosylation of BMP15 are properties that are unique to the pig in this study. The current study failed to detect native dimers secreted from the three species tested, despite evidence of formation of a GDF9 dimer within the red deer oocyte, although possible multimeric protein forms were detected. The detection of dimers in recombinant pig BMP15 and GDF9 suggests important differences between native and recombinantly-produced proteins.

## Materials and methods

### Oocyte collection

Ovaries of sheep (*Ovis aries*), red deer (*Cervus elaphus)* and pigs (*Sus scrofa domestica*) of unknown backgrounds were collected from local abattoirs and transported to the lab. Upon arrival at the lab, all ovaries were briefly washed in 70% ethanol and rinsed thoroughly with sterile saline.

Ovarian follicles were punctured using a 20-gauge needle to release the cumulus cell-oocyte complex (COC) into dissection media (M199 containing Earle’s salts, Sigma-Aldrich; supplemented with 0.1 g/L L-glutamine, 100 U/ml penicillin, 100 mg/ml streptomycin and 20 mM HEPES). The COCs were mechanically denuded by gentle and repeated drawing into and expulsion from a 5 mL syringe. The resultant cellular contents were passed through a 100 μM cell strainer (Falcon®; Corning®) for collection of oocytes. Pools of denuded oocytes exhibiting a robust zona pellucida were then transferred to microcentrifuge tubes containing fresh dissection media. The pooled oocytes were washed twice with dissection media using centrifugation steps (500* g* for four minutes) between washings to pellet the oocytes. After the final centrifugation step, the oocyte pools were either processed as oocyte lysate or CM samples. The numbers of pooled oocytes used for detection of GDF9 in oocyte lysates and in secreted media in sheep, pig and red deer were 100 for all species. The numbers of oocytes used for detection of BMP15 in sheep, pig and red deer oocyte lysates were 100, 80 and 100, respectively, and in secreted media were 125, 100 and 125, respectively.

For oocyte lysate samples, a protease inhibitor (Complete EDTA free protease inhibitor cocktail, Roche) was added at a final concentration of 1X and the samples were vortexed vigorously before centrifugation at 7000* g* for 1 min*.* The aliquots were then snap-frozen on dry ice and stored at -80 °C until further processing.

For CM samples, pools of oocytes were re-suspended in 25μL of incubation media (M199 containing Earle’s salts, Sigma-Aldrich; supplemented with 0.1 g/L L-glutamine, 100 U/ml penicillin, 100 mg/ml streptomycin and 26 mM NaHCO_3_) and placed in a 5% CO_2_ incubator at 95% humidity and at 39 °C for 18 h. Following incubation, the tubes were centrifuged at 500* g* for 4 min and the media was transferred into fresh 1.5 mL microcentrifuge tubes and oocytes were discarded. A protease inhibitor (Complete EDTA free protease inhibitor cocktail) was added to the CM at a final concentration of 1X and stored at  − 80 °C until further processing.

### Construction of expression plasmids

Recombinant proteins were generated to use as positive controls for Western blotting. Recombinant pig GDF9 and BMP15 was amplified from pig ovarian RNA by RT-PCR to generate full-length cDNA sequences. All primers were designed to incorporate restriction enzyme sites for sub-cloning purposes. The forward and reverse primer sequences for pig *GDF9* (NM_001001909) were 5’cctcgagactatggcgcttcccaga3’ and 5’gaattcttaacgacacgtgcactttg3’, respectively and for pig *BMP15* (HQ450759) were 5’gaattcgaacatgttgctgaacaagtctttc3’ and 5’tctagatcacctgcacgtacaggactgg3’, respectively. Recombinant red deer GDF9 and BMP15 was amplified from red deer ovarian RNA by RT-PCR to generate cDNA sequences from exon 2 (sequence information kindly provided by Dr Jenny Juengel; AgResearch Invermay, NZ). The forward and reverse primer sequences for red deer *GDF9* were 5’ctcgaggccatggcacttcccaacaaattc3’ and 5’gaattcttaacgacaggtacacttagtggcta3’, respectively and for red deer *BMP15* were 5’gaattccaagatggtcctcctgagcatcc3’ and 5’tctagatcacctgcatgtgcaggactgg3’, respectively. The PCR products were cloned into pGEMTeasy vector, which was then sub-cloned into the mammalian expression plasmid pEFIRES^49^ using *Xho1/EcoRI* sites for GDF9 and *EcoRI/XbaI* sites for BMP15*.*

### Production and analysis of secreted forms of recombinant proteins

The human embryonic kidney cell line (293HEK) was employed for single transfections of the GDF9- and BMP15-pEFIRES ligation products as previously reported^50^ using the FuGENE^®^HD transfection reagent (Promega) and following the manufacturers protocol. Transfected cells were plated into 75 cm2 culture flasks (Nunc; ThermoFisher Scientific) containing growing medium (Dulbecco modified Eagle medium, DMEM, supplemented with 10% (v/v) fetal calf serum, 100 IU/mL penicillin–streptomycin (Gibco) and 2 mM L-glutamine). Transfected cells were selected by puromycin (Sigma-Aldrich) resistance. Upon reaching confluency (every 3–4 days), the cells were passaged using warm trypsin (HyClone; ThermoFisher Scientific) and split 1:6. After cells reached confluency under 100 μg/ml of puromycin conditions, the growing medium was replaced with 12 mL of expression medium (50% DMEM with 50% Hams F12) supplemented with 100 IU/mL penicillin–streptomycin, with 2 mM L-glutamine, 0.01% bovine serum albumin (Sigma-Aldrich) and 100 μg/ml of heparin (Sigma Aldrich). The expression media containing the recombinant pig or red deer GDF9 and BMP15 proteins were removed, centrifuged at 1800* g* for 15 min and stored at  − 80 °C. These recombinant pig GDF9 and BMP15 and red deer GDF9 and BMP15 proteins will hereafter be referred to as *rec*pigGDF9, *rec*pigBMP15, *rec*deerGDF9 and *rec*deerBMP15, respectively.

### Enzymatic deglycosylation of BMP15 and GDF9

For de-glycosylation experiments, oocyte lysate and recombinant protein samples were first diluted to a volume of 14 µl each using dH_2_O. For recombinant proteins, 1 µl of HEK293-expressed *rec*pigGDF9 or *rec*pigBMP15 were used. For native proteins, 2 µl of 0.5 M sodium phosphate buffer (pH≈7.5), 2 µl of 10% NP40 (Sigma-Aldrich), and 2 µl of PNGase F (Promega) was added to each 14 µl sample. The samples were then incubated for 1 h at 37 °C. Western blotting was then performed under reducing conditions as described below.

### SDS-PAGE and western blotting

For SDS PAGE electrophoresis, separating and stacking gels containing 13.5% and 4% acrylamide, respectively were prepared using standard methods. Samples containing oocyte lysates, CM or recombinant controls were thawed. For each BMP15- and GDF9-labelled blot, 2 μL of *rec*pigGDF9 at a concentration of ~ 0.58 μg/mL (0.55, 0.61 ng/µL; 95% confidence interval) and 2 μL of *rec*pigBMP15 at a concentration of ~ 0.94 μg/mL (0.84, 1.09 μg/mL; 95% confidence interval) were made up to volume in incubation medium and added to separate lanes. These recombinant proteins were used as positive controls and swapped to use as negative controls. Sample volume was adjusted with incubation media to maintain consistency between wells. If a given sample was to be compared across several blots under different conditions, the samples were combined in one microcentrifuge tube, vortexed briefly, and then re-separated into individual tubes to ensure that the sample was as homogeneous as possible between blots.

For cross-linking studies, a freshly prepared BS3 (bis(sulfosuccinimidyl)suberate) crosslinking solution (ThermoFisher Scientific) was added to samples at a final concentration of 2 mM. Samples were then vortexed briefly and incubated at room temperature for 30 min to allow the non-cleavable crosslinking events to occur. Thereafter, a modified 2 × Laemmli loading buffer was added to each sample in a 1:1 ratio to achieve either non-reducing (0.033 M Tris–HCl pH6.8, 30% v/v glycerol, 3% w/v sodium dodecyl sulphate and 0.026% w/v bromophenol blue) or reducing (0.033 M Tris–HCl pH 6.8, 30% v/v glycerol, 3% w/v sodium dodecyl sulphate (SDS), 0.026% w/v bromophenol blue, 0.03 mM dithiothreitol and 0.1% 2-mercaptoethanol) conditions. Following denaturation at 95 °C for 5 min, samples were then centrifuged at 1000* g* for 2 min to remove any insoluble particles, and loaded onto stacking gels. For each protein in each species, samples were replicated at least three times under non-reducing, reducing and crosslinking conditions.

Each gel was placed in a Mini-Protean Tetra Cell running cassette (Bio-Rad Laboratories) and secured in place. The cell was filled with running buffer (1 M glycine, 0.12 M Tris and 0.1% w/v SDS), and the gels were electrophoresed for 1–1.5 h at 150 V. The proteins in the gels were then transferred to nitrocellulose membranes (Amersham Protran, GE Healthcare) in transfer buffer (0.025 M Tris, 0.038 M glycine and 20% v/v methanol) using a Criterion™ Blotter with plate electrodes (Bio-Rad Laboratories) according to manufacturer’s instructions.

Membranes were blocked with 5% (w/v) skim milk powder in blocking buffer (20 mM Tris–HCl (pH 7.5), 0.15 M sodium chloride and 0.1% v/v Tween 20). In each of the three species, GDF9 and BMP15 were detected using the monoclonal antibodies Mab37A and Mab61A. Both are mouse-generated monoclonal antibodies (IgG) which were made by Oxford Brookes University (UK) as previously described^51^. Mab37A was generated using full length *E. coli*-produced mature ovine GDF9 protein and has been shown previously to specifically bind ovine GDF9 and detect promature and mature forms^10,18,50^. Mab61A was generated against full length *E. coli*-produced mature ovine BMP15 protein and targets a 15 amino acid sequence towards the N-terminus of the mature peptide, SEVPGPSREHDGPES^50^. This region is predicted to be a flexible region with unknown function as previously described^15,52^. This region shares 100 and 93.3% identity with the red deer and pig sequences, respectively.

To test for antibody specificity, pre-adsorption experiments were performed on previously labelled blots. Before the pre-adsorbed antibodies were added, proteins were stripped from membranes through incubation in Stripping buffer (2% SDS, 0.06 M Tris–HCl pH 6.8, and 0.7% 2-mercaptoethanol) at 50 °C for 30 min with gentle agitation. E-coli-produced ovine BMP15 and ovine GDF9 was used for the pre-adsorption of Mab61A and Mab37A, respectively.

To investigate the reliability of the quantification and normalisation of the BMP15 and GDF9 mono-clonal antibodies (Mab61A and Mab37A, respectively), serial dilutions of recombinant proteins and/or oocytes were performed under reducing conditions (Supplemental Figs. 1 and 2).The relative band densities were determined using ImageJ Software (version 1.54f. with Java; National Institutes of Health) as previously described^53^. When bands were absent or when the band density fell outside of the linear range, the data point was omitted. The relative band densities were then converted into a proportion by dividing both the sample amount and relative band density to the most concentrated sample and band density for each protein type. Then, the relative proportion of sample was plotted against the relative density and linear regression analysis was performed. The r^2^ values were all > 0.90 and no statistical differences between the slopes were found, confirming that the semi-quantitative analyses performed on the Western blots presented herein were valid.

Chemiluminescent detection using WesternBright ECL-Spray (Western Blotting Detection System; Advansta) allowed visualisation of the membrane-bound labelled proteins and bands were imaged using an Omega Lum G Imaging System (Aplegen). Exposure lengths of one and ten minutes were used for GDF9 and BMP15, respectively. Semi-quantitative analyses of the relative band densities of proteins were performed under reducing conditions to disrupt any protein associations that might mask the absolute amount of the molecular forms present using ImageJ software (version 1.50b with Java; National Institutes of Health) as previously described^53^.

### Surface plasma resonance analysis of recombinant forms of GDF9 and BMP15

Due to an initial failure of *rec*deerBMP15 production, only *rec*pigGDF9, *rec*pigBMP15 and *rec*deerGDF9 were tested on their ability to interact with human (h) BMPR2 using SPR. A preparation of recombinant ovine GDF9 (*rec*ovGDF9)^29^ was included as a positive control given that ovine GDF9 has been shown to bind BMPR2 prior to recruitment of the Type 1 receptor^45^. As all recombinant proteins were in 293H-secreted media, preparations were partially purified by removal of all contaminants of < 10 kDa in molecular weight by dialysis. Proteins were loaded into dialysing tubing and placed in dialysing buffer (10 mM HEPES, 500 mM NaCl, 3.4 mM EDTA and 0.005% (v/v) Tween 20, pH 7.4) for 3 days, replacing the buffer daily. Partially purified proteins were then concentrated into a 600 μL volume by ultrafiltration using Vivaspin 6 centrifugal concentrators (Sartorius, molecular weight cut-off 10 kDa). The concentrated preparations of *rec*pigGDF9, *rec*pigBMP15, *rec*deerGDF9 and *rec*ovGDF9 were used immediately. As the amino acid sequence of the extracellular ligand-binding domain of the BMP type II receptor BMPR2 is almost invariant between human, mouse, porcine, ovine and cervine BMPR2, human (h) BMPR2 ectodomain protein was used for interaction analysis employing our established hBMPR2 protein production. The extracellular domain of hBMPR2 (amino acids 27 to 151) were produced with a C-terminal thrombin cleavage site (LVPRGS) and a His_6_-tag in baculoviral infected S*f*9 insect cells and purified as previously described^54^. The purified hBMPR2 receptor domain was *N-*biotinylated using EZ-Link sulfo-NHS-LC-biotin (Pierce) in 1:1.2 molar ratio as previously described^55^.

Surface plasmon resonance analyses were performed using a ProteOn XPR36 system (Bio-Rad). All measurements were performed at 25 °C using a flowrate of 100 µl/min and employing 10 mM HEPES, 500 mM NaCl, 3.4 mM EDTA and 0.005% (v/v) Tween 20 as running buffer. A GLC sensorchip (Bio-Rad) was activated N-hydroxysulfosuccinimide and N-(3-dimethylaminopropyl)-N-ethylcarbodiimide (Bio-Rad) following the manufacturer's recommendation and then coated with streptavidin to a density of about 2000 resonance units (RU, 1RU = 1 pg/mm^2^). Biotinylated hBMPR2 was then captured to a density of about 200 RU in one of the six flow cells of this streptavidin-coated sensorchip. Six consecutive log2 serial dilutions of the neat, partially purified GDF9 and BMP15 protein samples in HBST_500_ buffer were perfused as analytes over the above-described hBMPR2 sensorchip to yield SPR data for ligand-hBMPR2 interaction. Association was monitored for 120 s, dissociation was observed for 60 s. Data acquisition was performed in the so-called single-shot kinetics setup (biosensor rotated by 90° compared to ligand immobilization), which allowed simultaneous recoding of all six analyte concentrations. Regeneration of the biosensor was achieved with a 30 s perfusion pulse of 4 M MgCl_2_. Non-specific binding of the analyte to the sensor matrix and bulk face effects were removed from the raw SPR interaction data by subtracting the interaction of the analyte with an empty flow cell (coated only with streptavidin, but no ligand immobilized). Data were analysed with the software ProteOn Manager version 3.1 (Bio-Rad).

### Proliferation assays of recombinant proteins on sheep granulosa cells

Ovaries were trimmed of extraneous tissues and were transversely dissected at the hilum into two halves. The medulla region was removed, and the remaining cortical regions were washed in dissection media. Using a 21-gauge needle and 3 mL syringe, a cut in the surface of all visible follicles of healthy appearance was made and granulosa cells were flushed into the dissection media. All denuded oocytes and cumulus cell-oocyte complexes were removed, and the remaining cells were pelleted by centrifugation at 800* g* for 5 min. The cell pellet was re-suspended in 1 mL dissection media and viable granulosa cells were counted as determined using trypan blue exclusion. The cell suspension was pelleted again and washed once in McCoys incubation media (McCoys 5a media (Sigma) containing 2 mM glutamax, 0.3 mg/mL poly(vinyl alcohol), 26 mM NaHCO_3_, 100 IU/mL penicillin and 100 mg/mL streptomycin before being suspended in 1 mL McCoys incubation media. The 3H-thymidine incorporation assay was performed as previously described^50^, modified such that the total assay volume was reduced to 55μL. Each replicate experiment contained four wells of ovine granulosa cells that were incubated with 5 μL of un-purified expression media of H293 cells transfected with either PFIRES empty vector (Control), pig *GDF9* gene (*rec*pigGDF9), pig *BMP15* gene (*rec*pigBMP15), red deer *GDF9* gene (*rec*deerGDF9) or red deer *BMP15* gene (*rec*deerBMP15) expression media. Five replicate experiments were performed in total.

### Statistical analyses

Densitometry data was graphed and analysed, including linear regression analysis, using Prism Software (version 6.01; Graphpad). Band density analyses were only performed on samples processed under reducing conditions. Normalised average density values for the promature and mature bands in the sample lanes of each blot were calculated by dividing the measured density value of each band (measured in ImageJ) by the density of the mature band of the positive control of the same blot. This value was then adjusted to account for the different numbers of oocytes used for each species by dividing the value by the number of oocytes used in the sample lane and multiplying the result by 100.

Comparisons of normalised densitometry data from bands at the estimated size of mature and premature molecular forms, and the promature: mature form ratios were made across all four species in oocyte lysate and conditioned media, using a two-way ANOVA, followed by a Bonferroni’s post-hoc test if applicable (IBM SPSS Statistics). In all comparisons, *p* < 0.05 was considered significant.

### Supplementary Information

Supplementary Information.

## Data Availability

All data are contained within the manuscript.

## Abbreviations

BMP15: Bone morphogenetic protein 15
CM: Conditioned media
COC: Cumulus cell-oocyte complex
ER: Endoplasmic reticulum
GDF9: Growth differentiation factor 9
kDA: Kilodalton
OE: Oocyte CM
rec*deer*BMP15: Recombinant red deer BMP15 protein
rec*deer*GDF9: Recombinant red deer GDF9 protein
rec*pig*BMP15: Recombinant pig BMP15 protein
rec*pig*GDF9: Recombinant pig GDF9 protein
RER: Rough endoplasmic reticulum
SPR: Surface plasma resonance
TGF-β: Transforming growth factor β
