# Supplementary material for: Molecular forms of BMP15 and GDF9 in mammalian species that differ in litter size
Source: Sci Rep. 2023 Dec 16;13:22428. doi: 10.1038/s41598-023-49852-1 (PMC10725505; doi:10.1038/s41598-023-49852-1)
Supplement: Supplementary file 1 — Supplementary Information. [file 41598_2023_49852_MOESM1_ESM.docx]

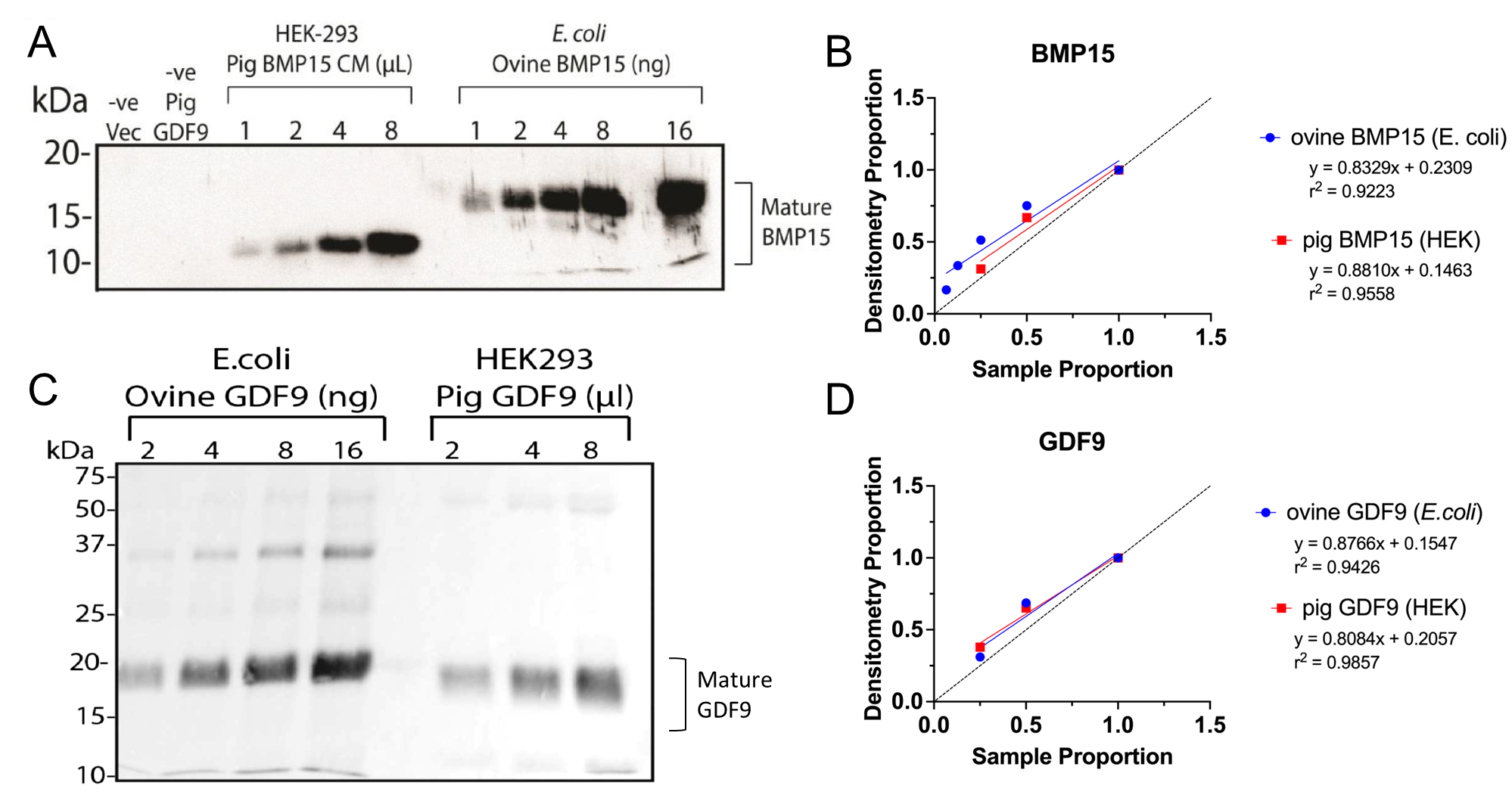
**Supplemental Figure 1** – Testing the reliability of the quantification and normalisation of the BMP15 band densities in BMP15 and GDF9 recombinant proteins. Immunoblots of serial dilution of HEK-293 or *E.coli* generated recombinant (A) BMP15 (Mab61A) and (B) GDF9 (Mab37A). (C) The relative proportion of sample quantity was plotted against the relative density proportion, as calculated by dividing the most highly expressed sample within the linear range. Linear regression analysis was performed, with the resulting slope equation and r^2^ value included. The black dotted line represents the predicted quantitative response whereby an increase in sample causes a proportional increase in the band density.


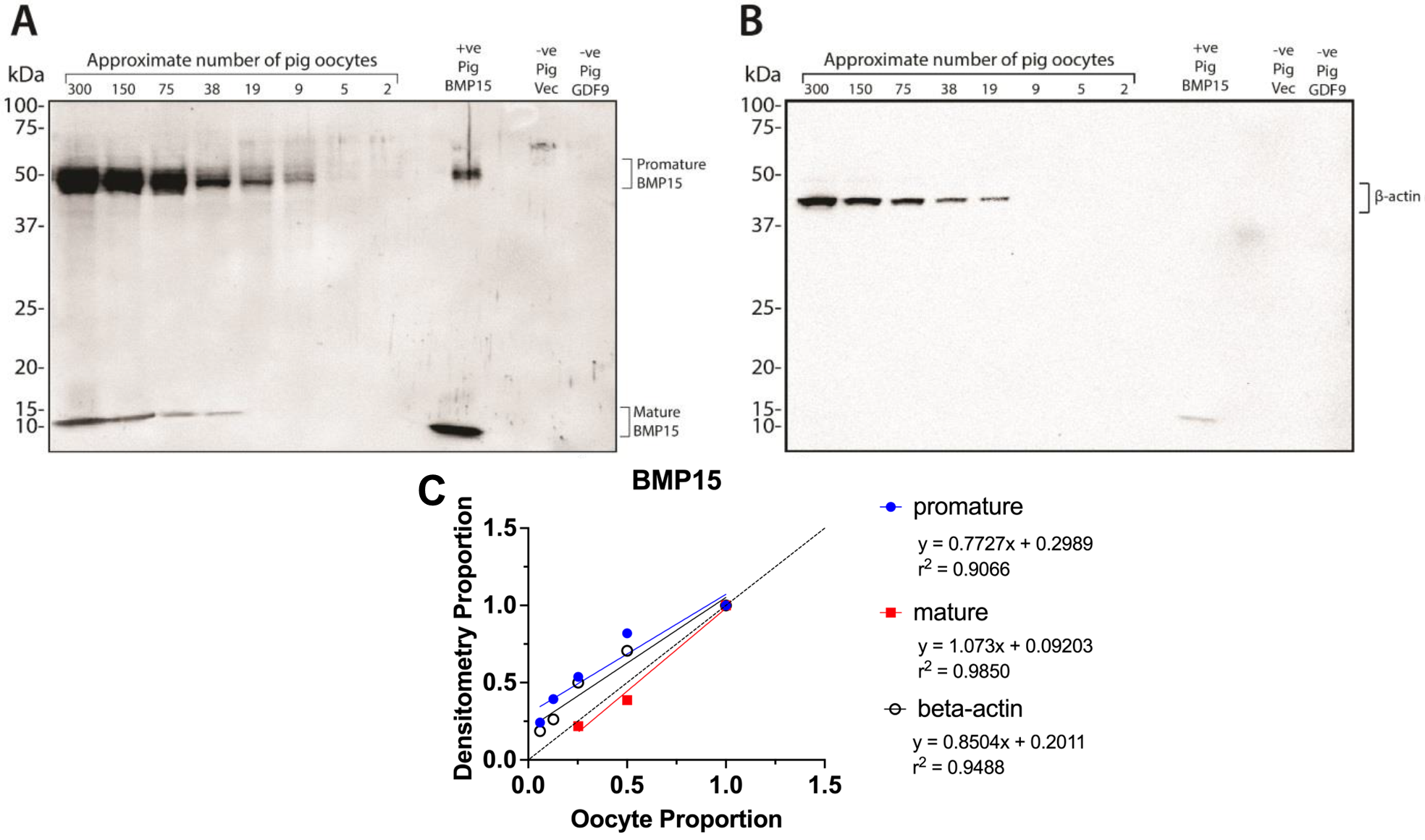
**Supplemental Figure 2** – Testing the reliability of the quantification and normalisation of the BMP15 band densities in oocyte lysates. (A) Immunoblot of a titration of pig oocyte lysates under reducing conditions for BMP15 (Mab61A) and (B) β-actin including pig BMP15 and GDF9 recombinant positive and negative controls, respectively. (C) The relative proportion of oocytes was plotted against the relative density proportion, as calculated by dividing the most highly expressed sample within the linear range. Linear regression analysis was performed, with the resulting slope equation and r^2^ value included. The black dotted line represents the predicted quantitative response whereby an increase in sample causes a proportional increase in the band density.


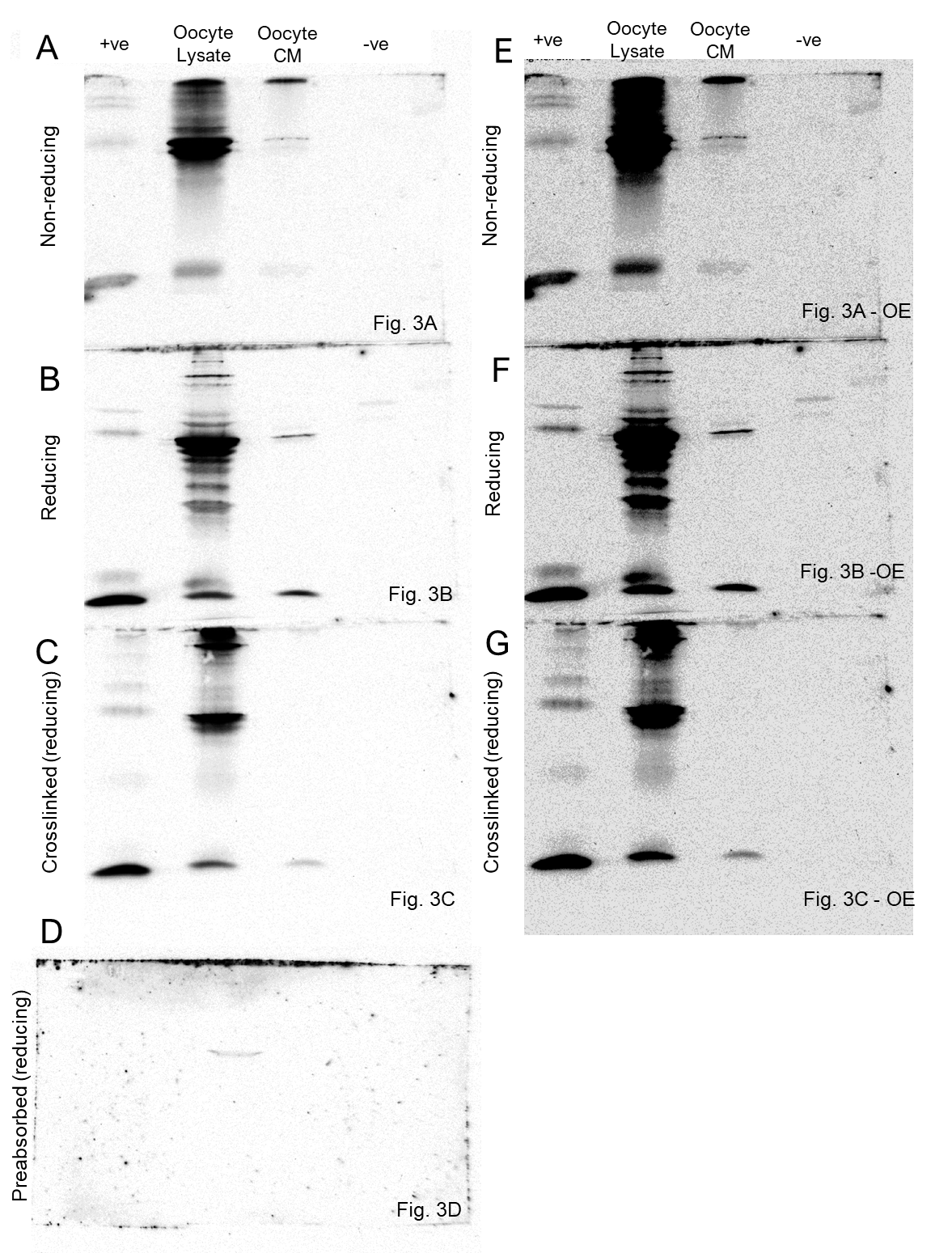


**Supplemental Figure 3** – (A,B,C,D) Uncropped full length Western blots associated with Figure 3 immunoblotted for ovine BMP15. Corresponding Figure 3 panels are written in lower right of image. (E,F,G) Identical overexposed (OE) full length blots corresponding to oocyte conditioned media (CM) OE lane in Figure 3 and to emphasize blot edges for compliance with digital image and integrity policies.


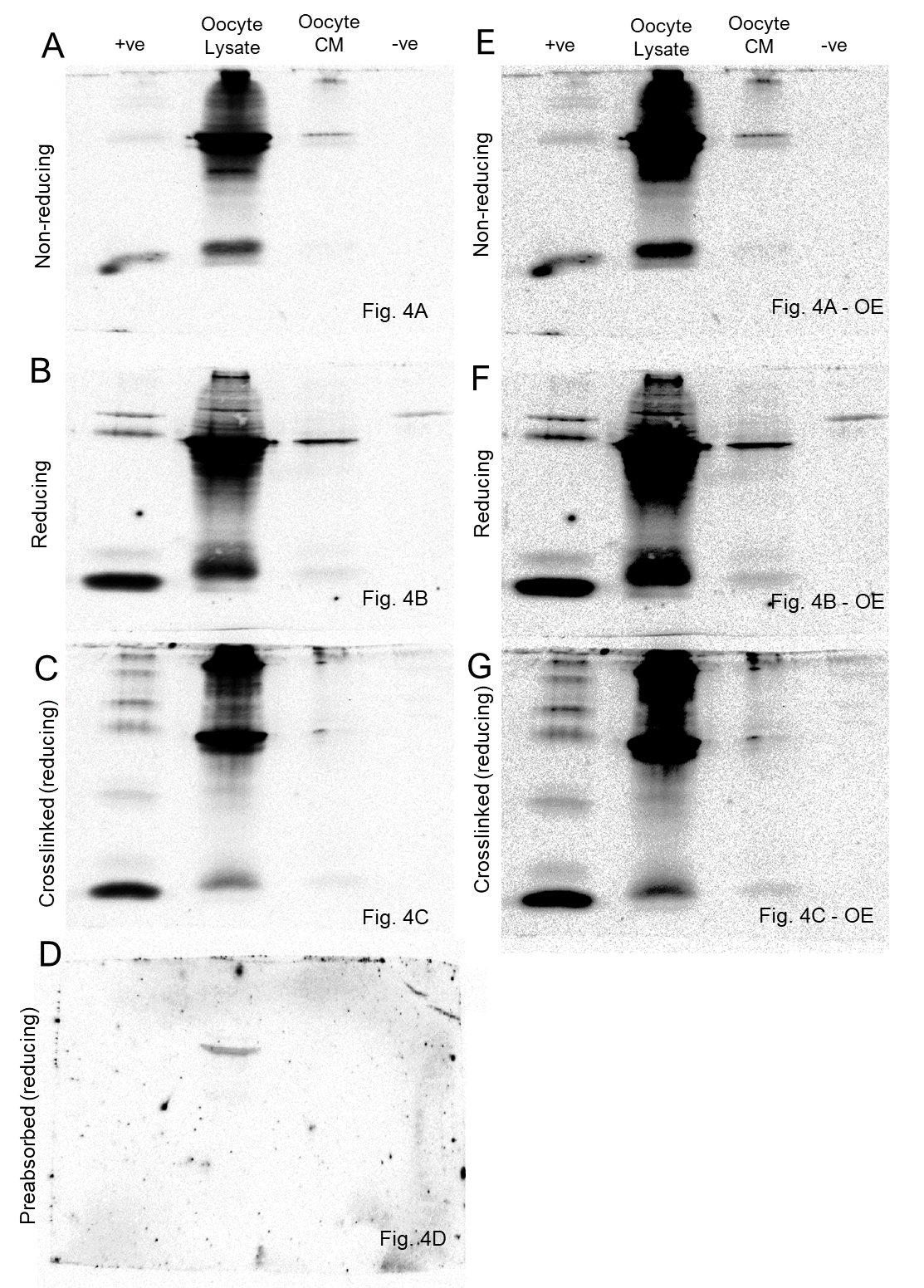


**Supplemental Figure 4** – (A,B,C,D) Uncropped full length Western blots associated with Figure 4 immunoblotted for red deer BMP15. Corresponding Figure 4 panels are written in lower right of image. (E,F,G) Identical overexposed (OE) full length blots corresponding to oocyte conditioned media (CM) OE lane (lane 3) in Figure 4 and to emphasize blot edges for compliance with digital image and integrity policies.


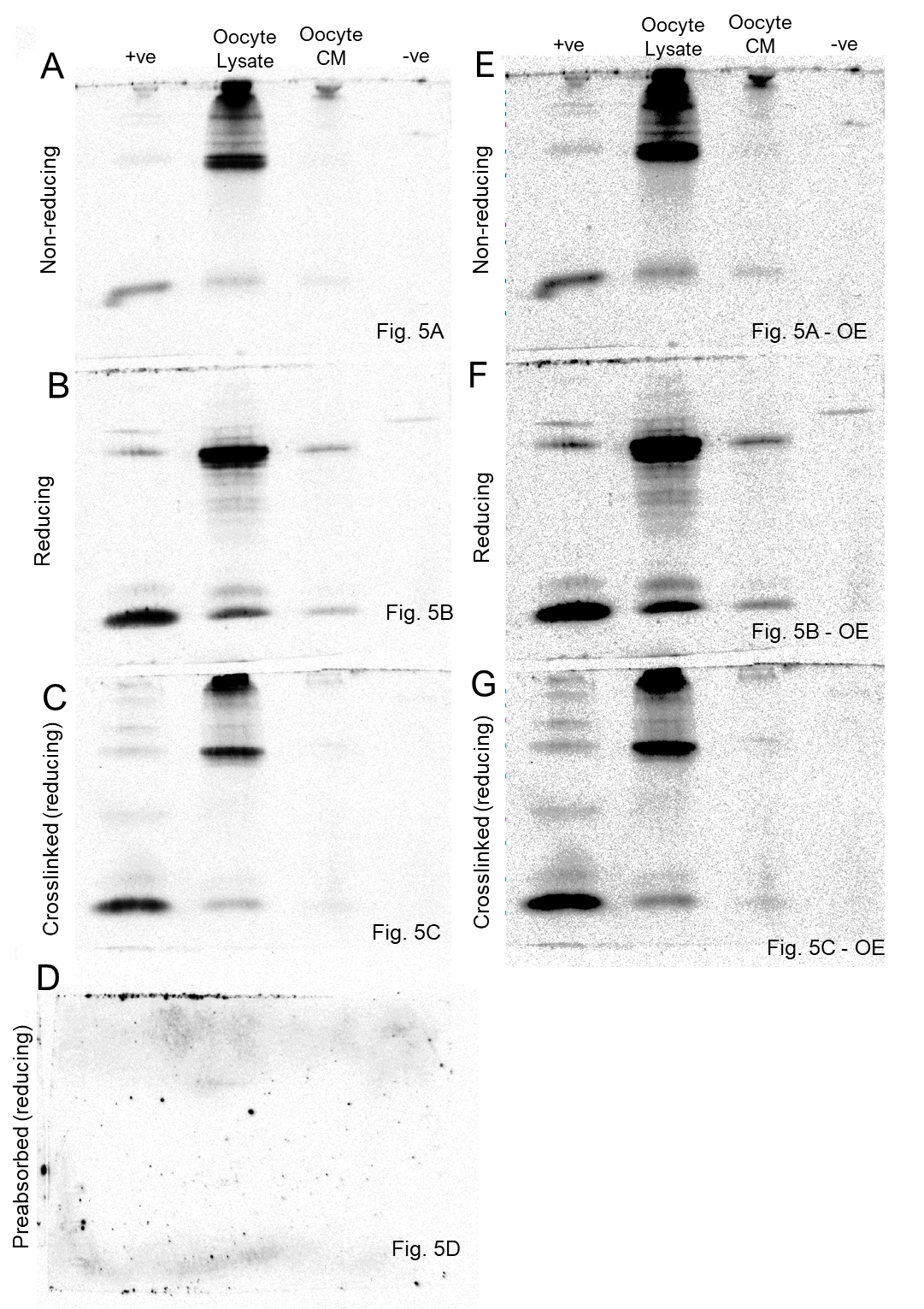
**Supplemental Figure 5** – (A,B,C,D) Uncropped full length Western blots associated with Figure 5 immunoblotted for pig BMP15. Corresponding Figure 5 panels are written in lower right of image. (E,F,G) Identical overexposed (OE) full length blots corresponding to oocyte conditioned media (CM) OE lane (lane 3) in Figure 5 and to emphasize blot edges for compliance with digital image and integrity policies.

**
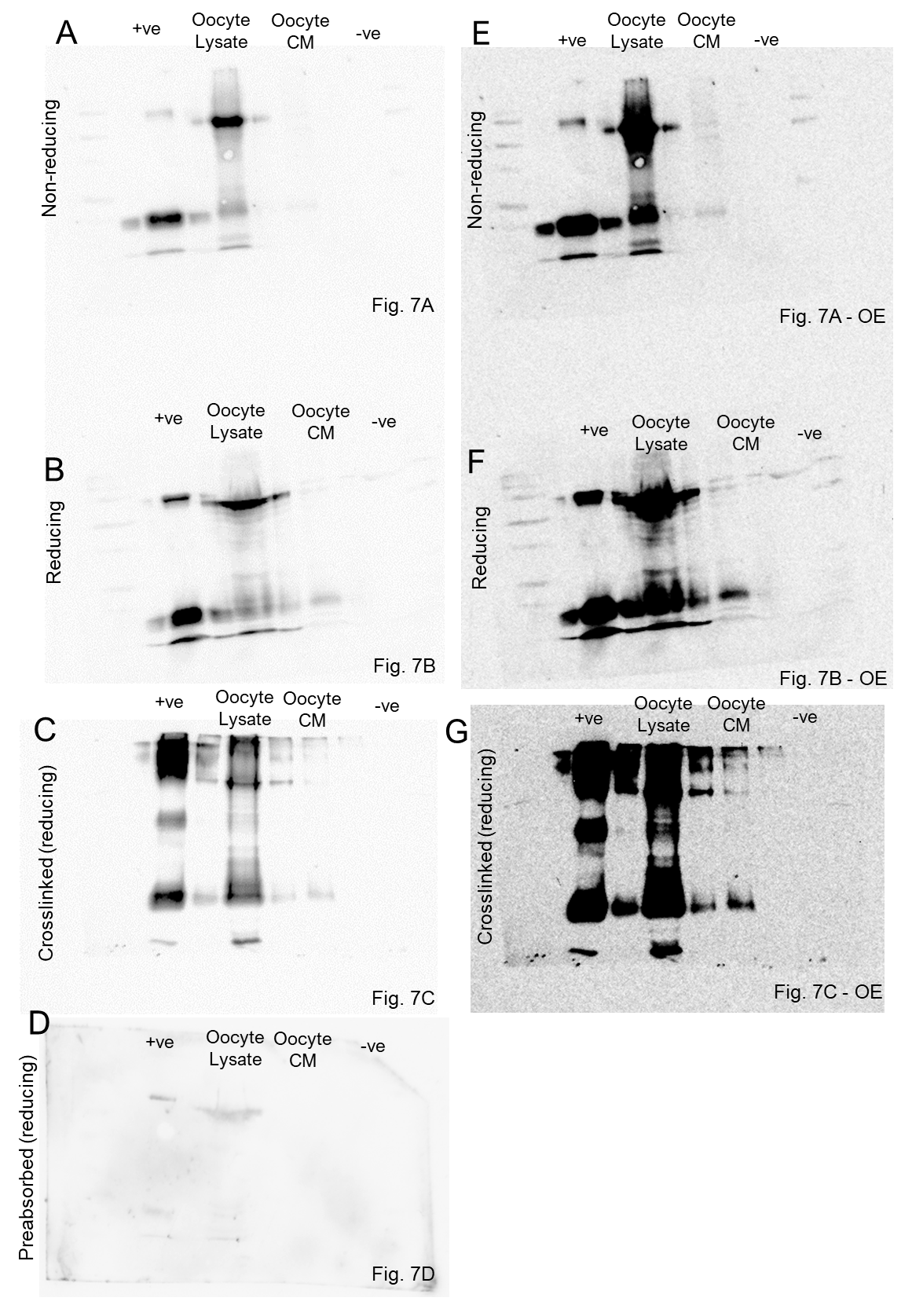
Supplemental Figure 6** – (A,B,C,D) Uncropped full length Western blots associated with Figure 7 immunoblotted for ovine GDF9. Corresponding Figure 7 panels are written in lower right of image. (E,F,G) Identical overexposed (OE) full length blots corresponding to oocyte conditioned media (CM) OE lane (lane 3) in Figure 5 and to emphasize blot edges for compliance with digital image and integrity policies.

**
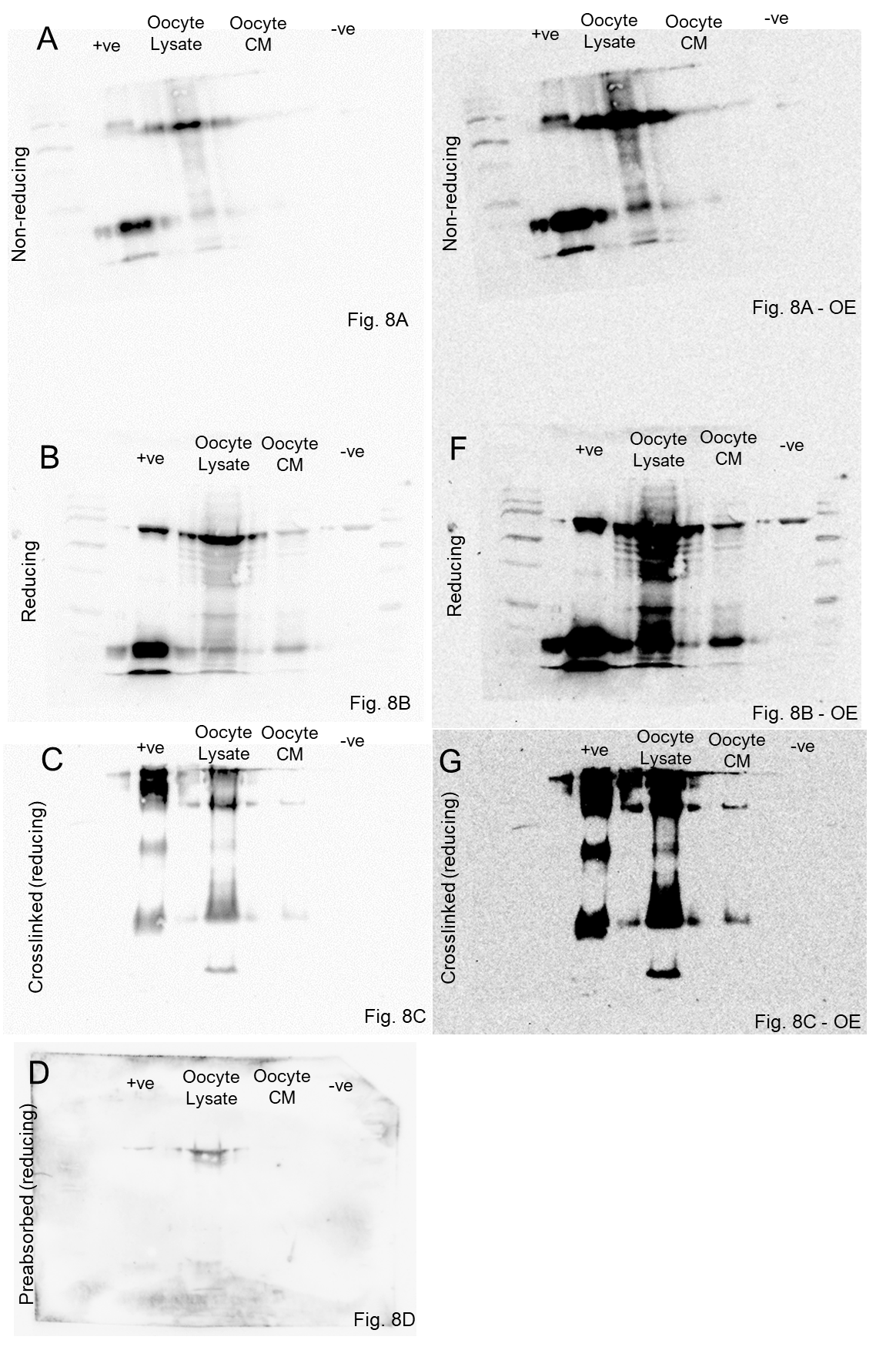
Supplemental Figure 7** – (A,B,C,D) Uncropped full length Western blots associated with Figure 8 immunoblotted for red deer GDF9. Corresponding Figure 8 panels are written in lower right of image. (E,F,G) Identical overexposed (OE) full length blots corresponding to oocyte conditioned media (CM) OE lane (lane 3) in Figure 8 and to emphasize blot edges for compliance with digital image and integrity policies.

**
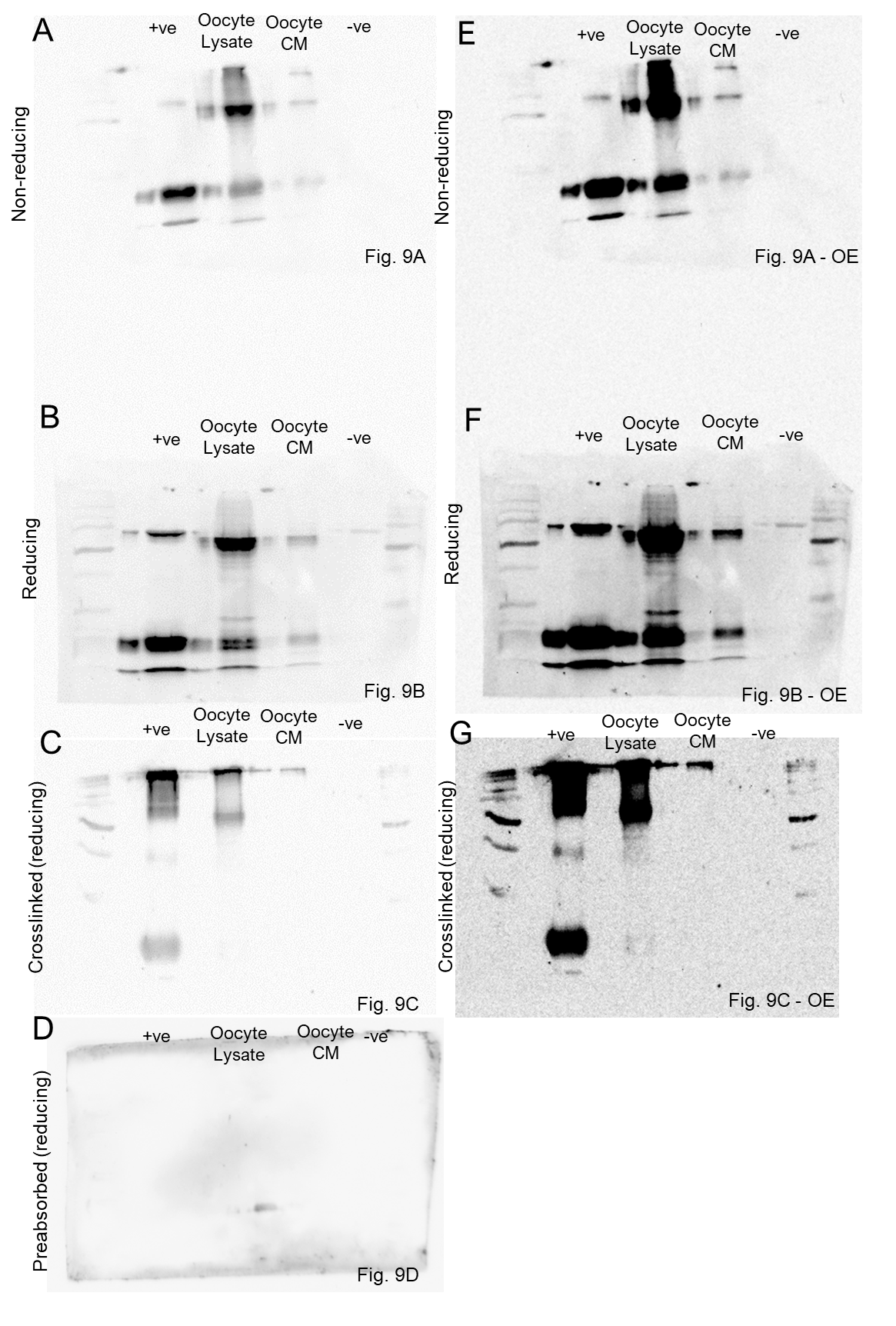
Supplemental Figure 8** – (A,B,C,D) Uncropped full length Western blots associated with Figure 9 immunoblotted for pig GDF9. Corresponding Figure 9 panels are written in lower right of image. (E,F,G) Identical overexposed (OE) full length blots corresponding to oocyte conditioned media (CM) OE lane (lane 3) in Figure 9 and to emphasize blot edges for compliance with digital image and integrity policies.


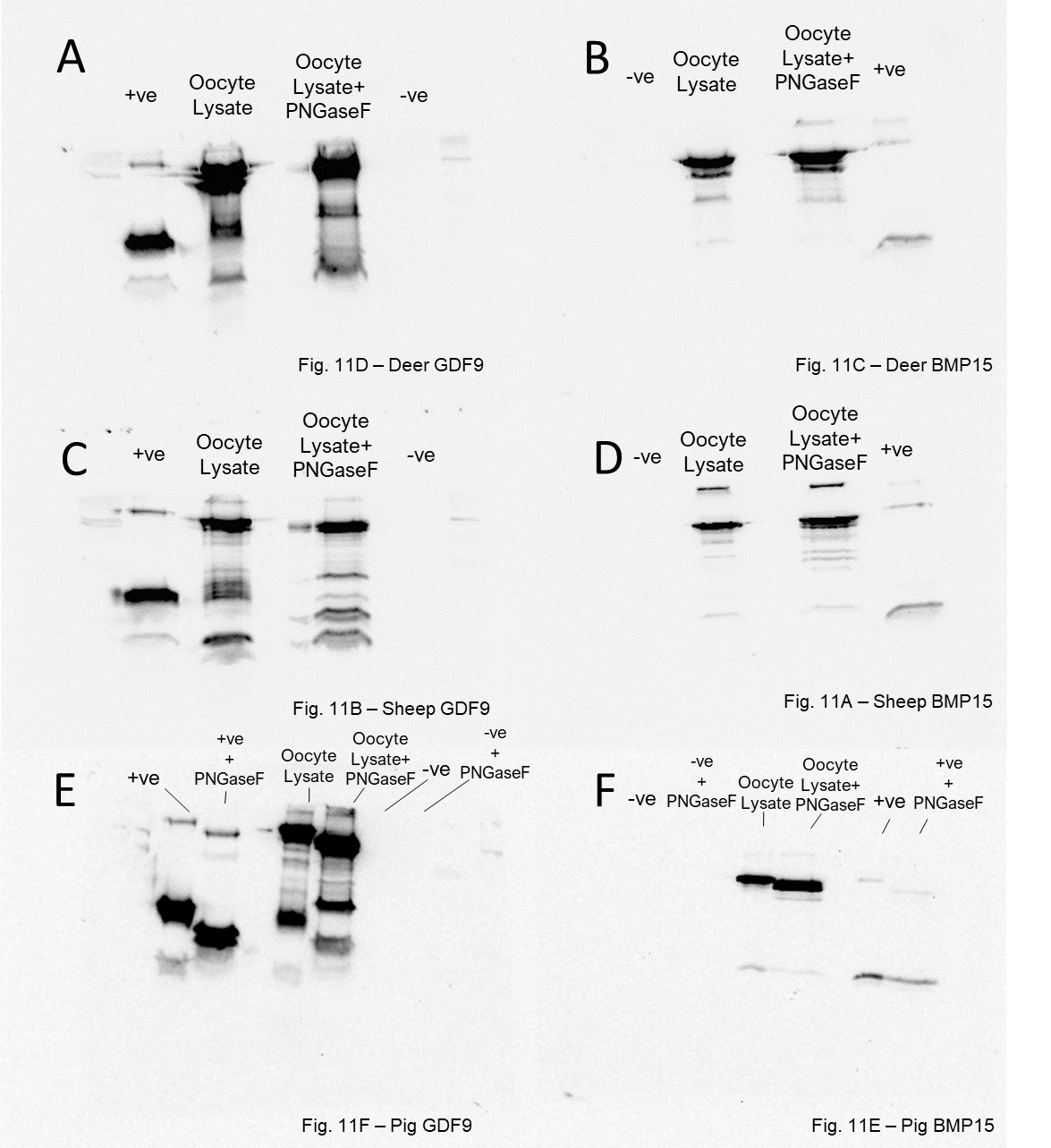


**Supplemental Figure 9** – Uncropped full length Western blots associated with Figure 11 immunoblotted for red deer, sheep, and pig GDF9 and BMP15 with or without PNGase treatment. Corresponding Figure 11 panels are written in lower right of image.


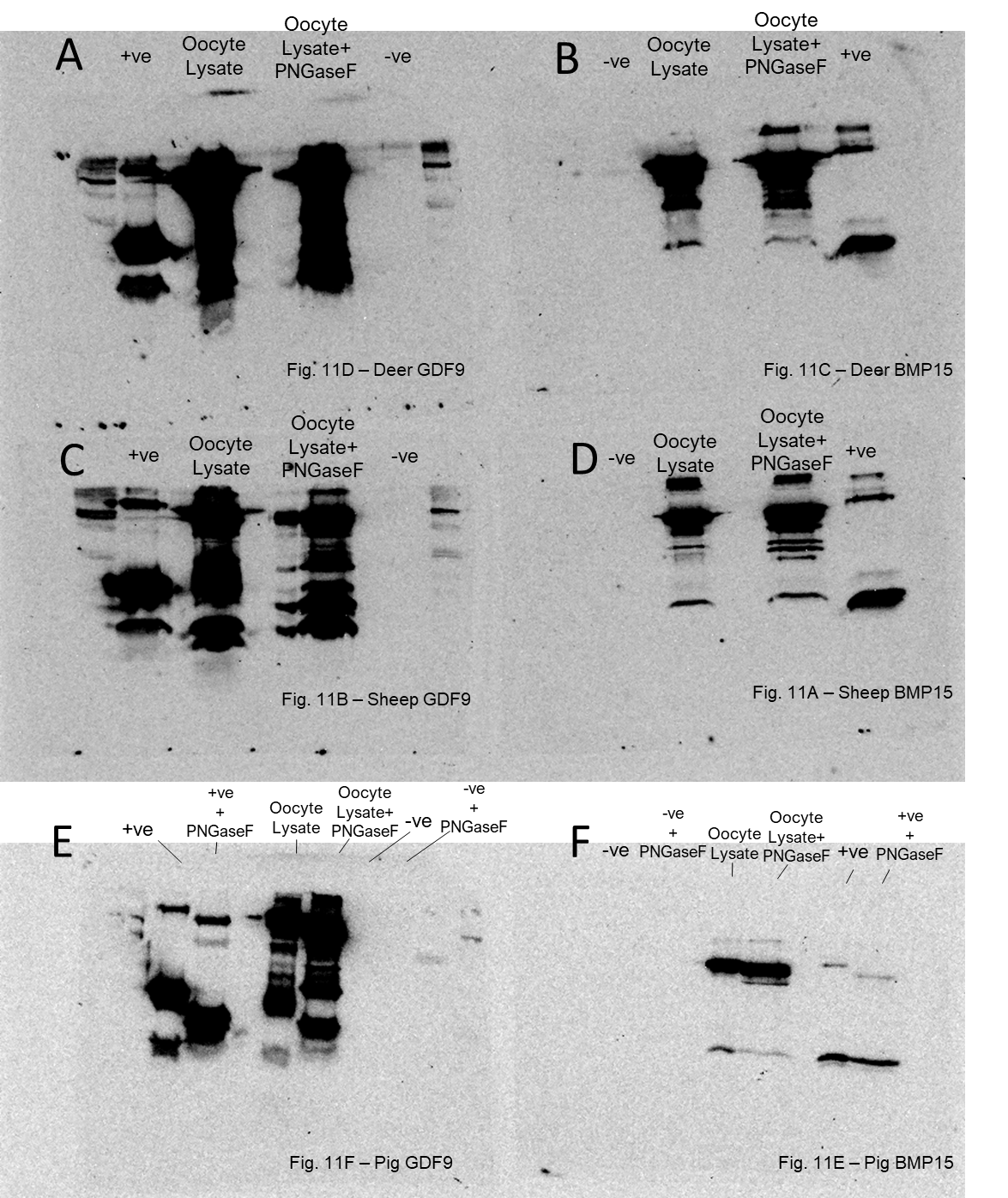


**Supplemental Figure 10** – Uncropped full length overexposed (to emphasize blot edges) Western blots associated with Figure 11 immunoblotted for red deer, sheep, and pig GDF9 and BMP15 with or without PNGase treatment. Corresponding Figure 11 panels are written in lower right of image.
